# Self-Reported Sleep Disturbance from Road, Rail and Aircraft Noise: Exposure-Response Relationships and Effect Modifiers in the SiRENE Study

**DOI:** 10.3390/ijerph16214186

**Published:** 2019-10-29

**Authors:** Mark Brink, Beat Schäffer, Danielle Vienneau, Reto Pieren, Maria Foraster, Ikenna C. Eze, Franziska Rudzik, Laurie Thiesse, Christian Cajochen, Nicole Probst-Hensch, Martin Röösli, Jean Marc Wunderli

**Affiliations:** 1 Federal Office for the Environment, 3003 Bern, Switzerland; 2 Empa, Swiss Federal Laboratories for Materials Science and Technology, 8600 Dübendorf, Switzerland; beat.schaeffer@empa.ch (B.S.); reto.pieren@empa.ch (R.P.);; 3 Swiss Tropical and Public Health Institute, 4051 Basel, Switzerland; danielle.vienneau@swisstph.ch (D.V.); maria.foraster@isglobal.org (M.F.); ikenna.eze@swisstph.ch (I.C.E.); nicole.probst@swisstph.ch (N.P.-H.); martin.roosli@swisstph.ch (M.R.); 4 University of Basel, 4003 Basel, Switzerland; 5 ISGlobal, Barcelona Institute for Global Health, 08003 Barcelona, Spain; 6 University Pompeu Fabra, 08002 Barcelona, Spain; 7 CIBER Epidemiologia y Salud Publica, 28029 Madrid, Spain; 8 Blanquerna School of Health Science, Universitat Ramon Llull, 08022 Barcelona, Spain; 9 Centre for Chronobiology, Psychiatric Hospital of the University of Basel, 4002 Basel, Switzerland; franziska.rudzik@posteo.de (F.R.); thiesse.laurie@gmail.com (L.T.); christian.cajochen@upk.ch (C.C.); 10 Transfaculty Research Platform Molecular and Cognitive Neurosciences, University of Basel, 4003 Basel, Switzerland

**Keywords:** noise exposure, sleep disturbance, exposure-effect relationship, traffic noise, noise effects, road traffic noise, railway noise, aircraft noise

## Abstract

This survey investigates the cross-sectional association between nighttime road, rail and aircraft noise exposure and the probability to be highly sleep disturbed (%HSD), as measured by self-report in postal and online questionnaires. As part of the Swiss SiRENE study, a total of 5592 survey participants in the entire country were selected based on a stratified random sample of their dwelling. Self-reported sleep disturbance was measured using an ICBEN-style 5-point verbal scale. The survey was carried out in four waves at different times of the year. Source-specific noise exposure was calculated for several façade points for each dwelling. After adjustment for potential confounders, all three noise sources showed a statistically significant association between the nighttime noise level LNight at the most exposed façade point and the probability to report high sleep disturbance, as determined by logistic regression. The association was strongest for aircraft noise and weakest for road traffic noise. We a priori studied the role of a range of effect modifiers, including the “eventfulness” of noise exposure, expressed as the Intermittency Ratio (IR) metric, bedroom window position, bedroom orientation towards the closest street, access to a quiet side of the dwelling, degree of urbanization, sleep timing factors (bedtime and sleep duration), sleep medication intake, survey season and night air temperature. While bedroom orientation exhibited a strong moderating effect, with an Leq-equivalent of nearly 20 dB if the bedroom faces away from the nearest street, the LNight-%HSD associations were not affected by bedroom window position, sleep timing factors, survey season, or temperature.

## 1. Introduction

Noise from road, rail and air traffic makes up the major part of total (technical) environmental noise, and it is well recognized that noise exposure has an important impact on public health [1]. The World Health Organization (WHO) estimated the burden of disease from environmental noise to be largely determined by noise-induced sleep disturbance, with more than 900,000 disability-adjusted life years (DALY) lost each year in western Europe due to noise at night, with road traffic being responsible for the largest fraction of this burden [2]. From a public health perspective, noise-induced sleep disturbance in and of itself is a relevant health outcome, but is also related to cardiovascular and metabolic morbidity [3]. Noise-induced sleep disturbance is suspected to be on the causal pathway to cardiovascular disease as non-habituating autonomic reactions to noise events may be important precursors of long-term cardiovascular outcomes [4,5,6]. There is ample evidence that nocturnal noise exposure leads to awakening reactions [7], elicits polysomnographically (PSG) measured arousals [8,9] and motility reactions [10,11], and leads to changes in sleep structure [12]. Sleep disturbances as a short-term outcome in the context of laboratory or observational small-scale field studies are often objectified as electroencephalographic (EEG) arousals or EEG awakening reactions. Self-reporting of noise-induced sleep disturbance is the measurement method of choice in large field surveys that assess the longer-term effect of noise in the context of people’s habitual environment. Despite the proliferation of original laboratory studies involving objective measures of noise effects on sleep in the last two decades, did measures of self-reported sleep disturbance inform the recommendations regarding nighttime noise exposure in the recently published WHO Environmental Noise Guidelines [1]. Reasons for this choice of outcome variable have been extensively discussed in [13].

While self-reported sleep disturbance can be measured in a variety of ways, including the use of multi-item questionnaire instruments, most population surveys that investigate noise effects measure noise-induced sleep disturbance with a simple questionnaire item. Most studies determine “high sleep disturbance” with the use of visual-analog scales and express the “percentage highly sleep disturbed” (%HSD) as the fraction of respondents that choose scale points close to the top of the scale, examples being [14,15,16].

Depending on the noise source and whether it is intermittent (such as aircraft overflights or train pass-by events) or rather continuous (such as traffic from a motorway), the effects of noise on sleep might either be better predicted by the number of noise events [17], their maximum sound pressure level and/or sound pressure level slope [7,10,18], or by average noise levels like the equivalent continuous sound pressure level (Leq) during nighttime (LNight). While energy-based exposure measures seem to predict noise annoyance reasonably well, they are less ideal to predict the impact of noise on physiological sleep quality indicators [19,20]. Exposure situations that are characterized by rather intermittent or relatively loud individual noise events feature more and longer noise-free intervals, but clearly also more pronounced single events that could trigger physiological reactions (e.g., heart rate or blood pressure increases) in the nighttime. The consideration of the intermittent characteristic of a noise exposure situation might therefore yield additional explained variance in statistical models on sleep disturbances, including self-reported symptoms.

To inform nighttime noise regulation policies for road, rail, and aircraft noise, this study investigated exposure-response relationships for %HSD based on a stratified random sample of the entire Swiss population exposed to at least one of the above three sources. It also aimed to elucidate the effect of noise intermittency [21] on %HSD, as well as to identify the effects of the following further potential effect modifiers: habitual bedroom window position, orientation of bedroom towards the nearest street, sound level difference between minimum and maximum façade point exposure, degree of urbanization, sleep timing (bedtime and sleep duration), sleep medication intake, survey season (winter, spring, summer, autumn), and night air temperature.

The survey was carried out in the framework of the interdisciplinary SiRENE project which aimed at investigating short- and long-term effects of transportation noise exposure on annoyance, sleep disturbances (self-reported as well as objectified in sleep laboratory experiments) and cardio-metabolic risks in epidemiological studies in Switzerland (for a review, see [22]).

## 2. Methods

### 2.1. Study Population, Design and Procedures

The study population comprised all officially registered residents in Switzerland in the age range between 19 and 75 years with known home address and known geographical location of their dwelling/apartment, including floor. From this population, a stratified random sample of residents all over the country was drawn based on exposure strata for road traffic, railway and aircraft noise. The stratification accounted for three sources (road, rail, air), noise exposure levels in two time periods (LDay (07−23 h) and LNight (23–07 h)), three categories of intermittency ratio IR [21] (see below) (low: 0−33%, medium: 33–66%, high: 66−100%), and ten 2.5 dB wide Leq exposure categories in the range between <45 dB(A) and >65 dB(A) for the day period (LDay), and between <35 dB(A) and >55 dB(A) for the night period (LNight). This resulted in a factorial design with 3 × 2 × 3 × 10 (= 180) cells. For each cell, we randomly selected 100 dwelling units and corresponding individual (postal) addresses. For the cell assignment, the sampling algorithm randomly decided if the façade point with the maximum or the minimum sound pressure level of a sampled dwelling unit determined cell membership. It is important to note that even if a dwelling unit was initially assigned for example to the source and time period “aircraft noise at day”, ultimately the exposure data for all sources and time periods at all façade points of the same dwelling unit were also obtained (if available).

For the survey, we implemented a mixed-mode approach using postal questionnaires and the option for completion of the survey online. Respondents automatically took part in a prize draw. To control for seasonal effects, the survey was carried out in four waves spaced three months apart. The bulk mailing dates of these waves were 18 Nov. 2014, 11 Feb. 2015, 08 May 2015, and 17 Aug. 2015. Non-responders were reminded one time with a reminder card sent approx. one month after the initial mailing. Shortly after the third wave, we conducted 224 computer assisted brief telephone interviews among randomly drawn initial non-responders to assess a potential non-response bias with respect to the distributions of noise exposure, age, noise annoyance, general noise sensitivity, pro-environmental attitude and education level. Further details regarding sampling strategy and survey procedure were reported previously in [23]. The study protocol was approved by the cantonal ethics commission of Bern (approval code KEK-Nr. 259/14).

### 2.2. Questionnaire Data

Self-reported sleep disturbance due to source-specific transportation noise was assessed using a 5-point verbal ICBEN (International Commission on Biological Effects of Noise)-style scale with the verbal marks “not at all”, “slightly”, “moderately”, “very”, “extremely” and the question “Thinking about the last twelve months at your home, during nighttime when you want to sleep, how much did <noise source> noise bother, disturb, or annoy you?”. Ticking one of the two top scale points (“very” or “extremely”, i.e., 40% of the scale length) defined “highly sleep disturbed” (HSD) status, abiding to the well-established convention originating from [24]. Further parts of the questionnaire were dedicated to personal data, the dwelling situation, specifically, the orientation of the bedroom towards the nearest street (directly facing the street, sideways to street, pointing away from street to a backyard or courtyard), sleeping habits, and prevailing bedroom window position in the three preceding months before the survey (closed, half-open or tilted, fully open). We also asked the respondents about the frequency of having taken prescription sleep medication in the four weeks preceding the survey (“never”, “less than once a week”, “once or twice a week”, “three or more times per week”).

We accounted for potential modifying effects of different sleep onset and rise times (estimated as weekly average from questionnaire answers) as well as for the weekly average duration of the time spent asleep. We calculated for each person the amount of minutes past 18:00 h before self-reported lights-off time. The higher this figure [in minutes], the later a person’s sleep period starts. We then assigned an “early”, “medium”, or “late” sleeper category to each person, based on a tercile split. We estimated a person’s sleep duration [in minutes] as period between estimated sleep onset (self-reported lights-off time + 15 min) and wakeup time (estimated self-reported rise time − 10 min). Again we assigned, by tercile split, “short”, “medium”, and “long” sleepers.

As sleep quality can be influenced by air temperature at night [25], and air temperature potentially triggers window closing behavior (independent from outside noise exposure but affecting indoor noise exposure), we obtained individual-level average night outdoor air temperatures derived from the respondent’s nearest official weather monitoring station (measured between sunset and sunrise) during the 90-day period preceding questionnaire completion.

The original questionnaires in German, French and Italian, as well as an English version are available in Supplementary File S2.

### 2.3. Noise Exposure Assessment

Noise exposure assessment procedures are described in a concise fashion in [23], and in more detail in [26,27]. All calculations were carried out for the reference year 2011 and encompassed all relevant transportation noise sources, i.e., all roads, railway lines and major airports in Switzerland. Noise exposure was calculated for all buildings with 1 to 3 receiver points per façade segment and floor (providing minimum and maximum sound exposure per dwelling unit). The noise exposure assessment for each façade point comprised yearly averages of the 1-hour-LAeq as well as the new metric Intermittency Ratio IR (see below). Based on this, source-specific LNight (LAeq from 23−07 h) and IR were calculated and assigned to the dwelling units.

The main analyses in this paper refer to LNight at the most exposed façade point. For road traffic noise, we also estimated bedroom outdoor and indoor LNight (i.e., in front of and inside the bedroom respectively). Such an approach was previously applied successfully in an epidemiological study on (indoor) noise exposure and hypertension [28], but was refined in several aspects here: To estimate the bedroom outdoor exposure level, we assigned the maximum (i.e., loudest, highest in level) façade point value of the dwelling unit if the bedroom window directly faced the street, and the minimum façade point value if it faced a backyard or courtyard. For bedroom windows facing sideways to the street, we assigned the maximum façade point value minus half the difference between maximum and minimum façade point value of the respondent’s dwelling unit. This gave us the estimated bedroom outdoor exposure level. From this value, a window position-dependent attenuation was subtracted to get the estimated indoor exposure level. Sound attenuation terms for open (10 dB), half-open/tilted (16 dB), and for closed windows (28 dB) were obtained by measurements carried out in a separate validation study in a subsample of 102 survey respondents [29].

The potential benefit of a (relatively) quiet bedroom regarding protection from road traffic noise can be expressed by the level difference between the estimated bedroom outdoor road traffic noise exposure and the energetic sum of the road + railway + aircraft noise exposure at the façade point where this sum is maximal (i.e., the loudest façade point of the dwelling with the highest noise level considering all sources together). The larger the resulting difference, the better protected the bedroom is relative to the dwelling unit as a whole from nearby road, railway and aircraft noise. In consequence, we hypothesized that the larger differences should relate to smaller %HSD. We thus estimated a level difference indicator *D_quietside* to parametrize this difference.

To reflect the intermittent nature of a noise exposure situation (and to investigate its impact on sleep disturbance), we used the acoustical descriptor IR, whose calculation principle is described in [21]. IR expresses the energetic contribution of individual noise events from a specific noise source relative to the total sound energy (produced by all noise sources together) in a given time period. An IR of higher than 50% means that more than half of the total sound energy is caused by “distinct” noise events (which are defined using a dynamic level threshold). Situations where events clearly emerge from background noise (e.g., at a receiver point near a railway track) yield IR values close to 100% whereas constantly flowing traffic situations with a steady noise level, e.g., from a distant motorway, only yield small IR values. In the present study, we determined IR on the LNight-corresponding façade point in the time period between 23 and 07 h, henceforth referred to as *IRNight*.

### 2.4. Statistical Analysis

Individual noise exposure at the minimum and maximum façade point was reassigned based on the correct floor information indicated by the respondents, where necessary. Those cases with no calculated exposure or with less than 20 dB(A) LNight exposure for a given source at the maximum façade point were excluded from analyses involving that source (to avoid that persons essentially unexposed to a particular source unjustifiably impact on the calculation of exposure-response models for that source). To derive exposure-response relationships for the outcome %HSD, we regressed the probability of being highly sleep disturbed (P_HSD_) on outdoor LNight at the most exposed façade point of the dwelling unit of the respondent.

Exposure-response models were developed using logistic regression and were calculated separately for each noise source (road, rail, or air). Both crude and adjusted models were established. The adjusted statistical modeling pursued a ‘risk factor approach’ that primarily focused on the unbiased modeling of the slope coefficient of LNight. The adjusted models were controlled for the *a priori*-selected predictors sex, age, interview mode (postal or online), and language of interview (German vs. French or Italian). This list of explanatory variables in the adjusted model was deliberately kept short, as experience showed that adjusting for a large range of predictors could result in over-adjustment (e.g., when adjusting for noise coping behavior or noise sensitivity), attenuating the real effect of the noise exposure, or inflate a modeled exposure-response relationship. The variables in the adjusted model were checked for nonlinear associations with the outcome %HSD using fractional polynomials, confirming for all noise sources that no transformations were necessary. Multicollinearity between variables in the adjusted model was evaluated by means of calculating variance inflation factors (VIF). Interaction terms were not included in the (main) adjusted exposure-response model (see Section 3.2 “Exposure-response relationships”), however, we (separately) investigated effect modification via stratified categorical analysis of the following potential effect modifiers, in interaction with LNight: IR (categorized as “low” (<33%), “medium” (33%−66%), “high” >(66%)), habitual bedroom window position (open, half-open/tilted, closed), orientation of the respondent’s bedroom towards the nearest street (away from street, sideways to street, pointing towards street), level difference indicator D_quietside (difference categories: <5 dB, 5−10 dB, 10−20 dB, >20 dB), degree of urbanization of the respondent’s municipality, as expressed by Eurostat’s DEGURBA indicator [30], bedtime category (“early” (<22:53 h), “medium” (22:53–23:31 h) or “late” (>23:31 h) sleepers, determined by tercile split), sleep period duration (“short” (<7.2 h), “medium” (7.2−8 h), or “long” (>8 h) sleepers, determined by tercile split), sleep medication intake in the four weeks preceding the survey (“yes” vs. “no”), season (winter, spring, summer, autumn), and night air temperature tercile split categories (<7 °C, 7−11 °C, >11 °C). Effect modification was investigated in the full sample where the adjusted model additionally included the categorized effect modifier in question and its interaction with LNight (to allow for non-parallel exposure-response curves). Tests of significant differences between specific levels of the effect modifier in question (paired comparisons) were accomplished with Tukey’s HSD post hoc test. Results of the effect modification analyses are jointly tabulated in Table S1 in Supplementary File S1.

It was clearly established recently, that the more spatially fine-grained the noise exposure assessment is (e.g., façade points vs. noise exposure grids), the steeper exposure-response relationships become, at least pertaining to cardiovascular outcomes [31]. This raises the question, which measurement/calculation location of the pertinent noise exposure provides the best %HSD predictions. Most often, in studies of noise-induced sleep disturbances due to noise exposure, the reported noise metric refers to LNight at the most exposed façade of the dwelling (or whole building), but not the bedroom, even if the latter would provide a more meaningful exposure measure for the time period when respondents sleep. We thus argue that there are good reasons to explore other noise metrics in relation to %HSD: It may be that respondents have in mind very specific time periods in thinking about experiences with noise-disturbed sleep; or, the sleep disturbance response may not develop due to nighttime noise exposure, but rather from daytime exposure. To reveal which of the many possible noise metrics best predicts self-reported noise-induced sleep disturbance in this survey, we calculated explained variance and model fit statistics for in total 18 (20 for road traffic noise) different exposure metrics. More specifically, we considered the average Leq in the time periods 19−23 (evening), 23−01 (early night), 01−05 (core night), 05−06 and 06−07 h (morning), in addition to Lden, LDay, Leq24, and LNight, all at the minimum and maximum façade point, and in the case of road traffic noise, the estimated bedroom outdoor and indoor LNight. We determined for each of these exposure variables the Akaike (AIC) and Bayesian (BIC) Information Criterion, pseudo R square values (Nagelkerke and McKelvey & Zavoina), as well as the log-likelihood and deviance by replacing LNight with the exposure variable in question in the adjusted model. Statistical significance was considered at an alpha level of 0.05. Inferential statistical analyses were performed using the procedures ‘glm’, ‘lme4 glmer’, ‘mfp’, ‘emmeans’, ‘car vif’, ‘smatr sma’ and ‘standardize’ in R version 3.5.1 (R Foundation for Statistical Computing, Vienna, Austria).

## 3. Results

### 3.1. Response and Exposure Distribution Data

From the original 18,000 questionnaires/cover letters sent out, 5592 were returned (31% response rate). 21.34% of the respondents completed the questionnaire online. General response statistics are given in Table 1. A detailed breakdown of the response rates by noise source, time period, LDay category, LNight category, and respective IR category as well as results of the non-response analysis are provided in [23].

**Figure 1:**
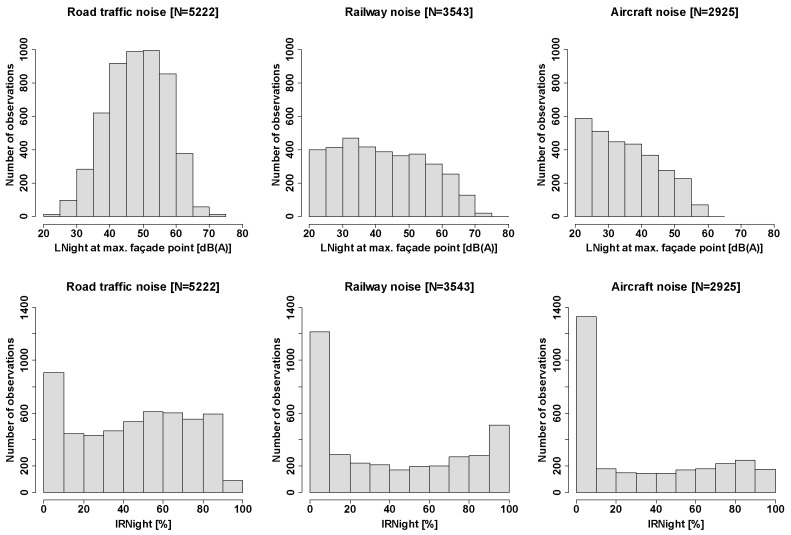
Frequency distribution of the LNight **(top)** and IRNight **(bottom)** among all valid cases. IRNight (bottom) is calculated at the corresponding façade point with the maximum LNight exposure for each respondent.

Figure 1 displays frequency distributions of outdoor LNight at the maximum façade point and corresponding distributions of IRNight. The quite large number of cases in the lowest-IR category for railway and aircraft noise is owed to the fact that IRNight = 0% was assigned to receiver points with very low or unavailable exposure data (which was more common for railway and aircraft noise).

**Figure 2:**
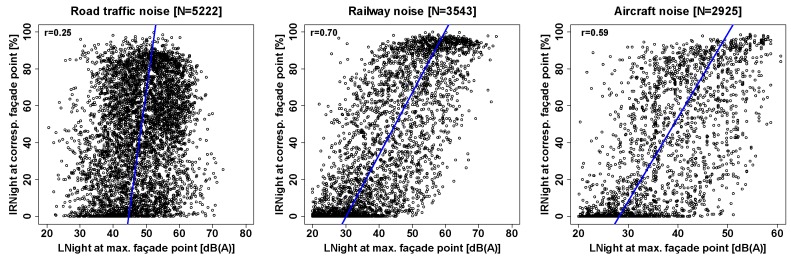
Scatterplots incl. total least squares (i.e., ‘orthogonal’ or ‘major axis’) regression line (blue) of LNight vs. façade-point-corresponding IRNight for road traffic, railway, and aircraft noise exposure in the present sample. Corresponding correlation coefficients are given in the top left corner of each plot.

The (as large as possible) decorrelation of IR from the Leq is an important feature of a noise metric that aims at explaining additional variance of noise effects which is not captured in the Leq (or here, more specifically, the LNight). Therefore we inspected the association between IRNight and LNight, which is shown in scatterplots in Figure 2. As one can see, the correlation was relatively weak for road traffic noise, whereas for the clearly “event-ish” noise sources railways and aircraft, IRNight and LNight correlated more strongly. Table 2 lists observed %HSD for each LNight category (in 5 dB steps) for the maximum façade point.

### 3.2. Exposure-Response Relationships between LNight and High Sleep Disturbance (%HSD)

Coefficients of the crude and adjusted models for the association between exposure to LNight at the maximum façade point and %HSD are presented in Table 3. Its results show LNight to be a statistically significant predictor for %HSD in both the crude and adjusted model, with aircraft noise having the strongest effect, and road traffic noise the weakest.

**Figure 3:**
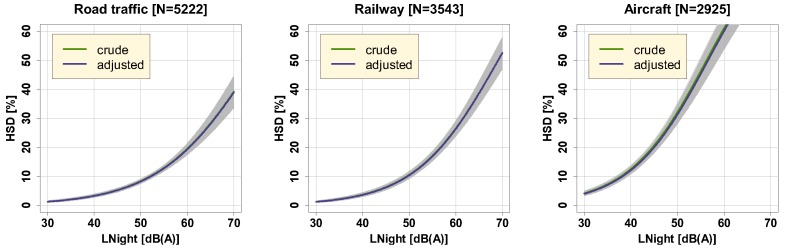
%HSD as function of LNight at the maximum façade point for road traffic, railway, and aircraft noise. The curves reflect both the crude and adjusted model with covariates centered on the mean (i.e., for the road traffic model: male sex = 0.47; age = 49; German language = 0.66; interview mode postal = 0.78 – for the railway model: male sex = 0.49; age = 48; German language = 0.69; interview mode postal = 0.77 – for the aircraft model: male sex = 0.47; age = 48; German language = 0.74; interview mode postal = 0.76.). The curves include 95% (overlapping) confidence intervals as shaded areas.

Figure 3 displays exposure-response curves for both the crude and the adjusted model. For the adjusted curves, all covariates were centered on the mean. Due to almost identical coefficients of the LNight effect, both crude and adjusted exposure-response curves were almost congruent, thus barely distinguishable.

In order that the exposure-response relationships emanating from our data can directly – rather than via using generic conversion terms—be applied to other time periods/noise metrics, the coefficients of the logistic equations for LNight as based on the time period of 22−06 h and 23−07 h, as well as for the Lden (using the partitioning 07−19 h (day), 19−23 h (evening), and 23−07 h (night)) are reported in Table 4. Coefficients are provided for both the crude (left columns) and adjusted model (right columns).

### 3.3. Effect Modification

In the following, exposure-response curves that account for a range of potential effect modifiers are presented. To easily grasp the relevance of each effect modifier, we show all exposure-response graphs, even if the respective effect modifiers were not statistically significant. Coefficients of the full sample models (whose curves are presented in the following) are jointly tabulated in Table S1 in Supplementary File S1. Each subchapter starts with a short introduction about why each effect modifier is specifically looked at.

#### 3.3.1. Eventfulness of Noise Exposure as expressed by the Intermittency Ratio (IR)

**Figure 4:**
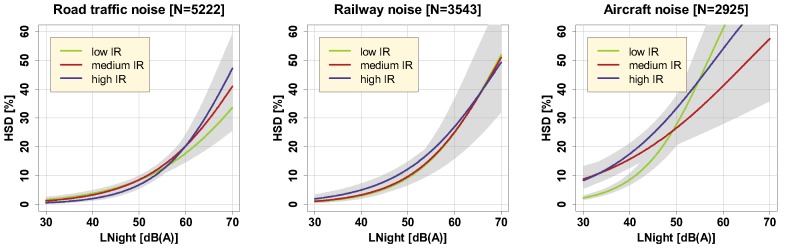
Exposure-response relationships for road traffic (left), railway (middle), and aircraft noise (right) in the full sample for three different IRNight categories, including 95% (overlapping) confidence intervals (shaded areas). Curves are based on the adjusted model in the full sample with covariates centered on the mean.

In the SiRENE study thus far, IR was demonstrated to act as a relevant effect that modifies the road traffic noise exposure-response relationship, in particular, for the following outcomes: Percent highly annoyed (%HA) (lower %HA with higher intermittency, [23]); cardiovascular disease mortality (reversed U-shaped relationship with higher disease risk for medium intermittency, [33]); arterial stiffness (increased in highly intermittent (IR > 80%) nighttime noise environments, [34]); and efficiency of recovery from noise-disturbed sleep (quicker recovery at low intermittency, [35]). Inspired by the latter finding, we hypothesized that rather “eventful” noise exposure situations with clearly distinguishable noise events in the night would more often trigger (acute) sleep disturbances than exposure to continuous noise. Figure 4 illustrates how the relationship between LNight and %HSD is affected by different levels of IRNight.

Results revealed that noteworthy effects of IR appear with road traffic noise at (particularly) higher LNight levels, and with aircraft noise up to about 50 dB(A), where in both cases, according to expectation, noise scenarios with higher IR were associated with higher %HSD. The effects of the IRNight category were significant for both these sources (cf. Table S1 in Supplementary File S1). Paired comparisons (contrasts) between factor levels of the IR category were significant in the case of aircraft noise low IR vs. medium IR (−1.0412 on the log odds ratio scale, *p* < 0.0001, Tukey-adjusted) and aircraft noise low IR vs. high IR (−1.0794 on the log odds ratio scale, *p* < 0.0001, Tukey-adjusted). For aircraft noise, situations with low IR thus seemed to be significantly less sleep disturbing at low LNight levels. A slightly stronger effect of high IR as compared to low and medium IR could also be seen with railway noise up to about 60 dB, although this effect was not significant.

All in all, the clearest modifying effect of IR was seen for road traffic noise levels above about 60 dB(A)—an effect which pointed to the expected direction, namely, that under such exposure conditions, situations with high IR, i.e., with dominating single events, cause higher sleep disturbance. In interpreting these results, it is relevant to be aware of the relatively small number of cases with high LNight (i.e., > 60 dB(A)) that were obtained by railway and aircraft noise on the one hand, and on the other, that IR was considerably correlated with LNight regarding railway and aircraft noise (cf. Figure 2).

#### 3.3.2. Bedroom Window Position at Night

Lercher and colleagues could show that the risk for hypertension was increased in those who slept with open windows during the night, but decreased in those where the bedroom was not facing the main road [36]. Similarly, Babisch found higher cardiovascular risk estimates for people habitually sleeping with windows open [37]. Foraster et al. observed more consistent associations between indoor noise levels in the bedroom (after considering bedroom window position and bedroom orientation) with hypertension and high systolic blood pressure, than with outdoor exposure at the address façade [28].

**Figure 5:**
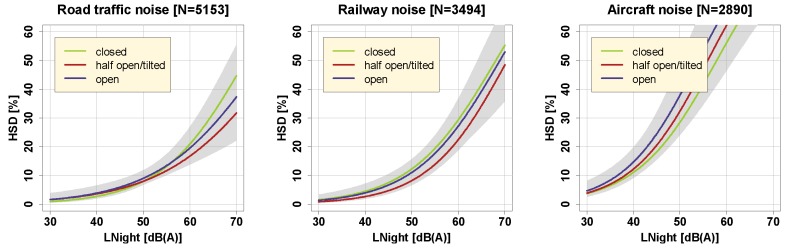
Exposure-response relationships for road traffic (left), railway (middle), and aircraft noise (right), for different habitual bedroom window positions (closed, half-open/tilted, open), including 95% (overlapping) confidence intervals (shaded areas). Curves are based on the adjusted model in the full sample with covariates centered on the mean.

Figure 5 shows exposure-response relationships in the present study separately for those who sleep with closed, half-open/tilted, or fully open bedroom windows. As evidenced in Figure 5, window position did not seem to affect exposure-response relationships for %HSD in a systematic (significant) way (cf. Table S1 in Supplementary File S1). One would expect that people who close their windows would reduce their indoor noise levels and hence be *less* sleep disturbed – but the opposite could also be true, namely that those (or at least some) who close their windows due to noise are particularly disturbed, which would, as a matter of reverse causality, be reflected in *higher* self-reported sleep disturbance. It is well possible that the two effects balance each other out and thus curves come to lie close together.

#### 3.3.3. Bedroom Orientation towards Closest Street

**Figure 6:**
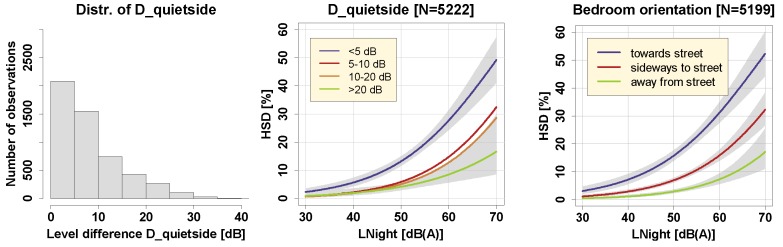
Left: Frequency distribution of the level difference indicator D_quietside. Middle: Exposure-response relationships for road traffic noise for four groups of D_quietside; 95% confidence intervals as shaded areas shown for the <5 dB and >20 dB category only. Right: Exposure-response relationships for road traffic noise for different orientations of the bedroom window, including 95% confidence intervals as shaded areas. LNight always refers to the maximum façade point of the dwelling unit. Curves are based on the adjusted model in the full sample with covariates centered on the mean.

Babisch et al. showed 20 years ago that road traffic noise exposure increases the risk of cardiovascular disease more in those whose bedroom is oriented towards the road [37]. Moreover, Foraster et al. [28] observed more consistent associations between road traffic noise and both hypertension and high blood pressure when estimating road traffic noise at the specific bedroom façade. We hypothesized that people having their bedroom away from the nearest road or street should potentially be less disturbed in their sleep. This hypothesis was investigated in three groups of respondents: those who indicated that their bedroom window was pointing towards/facing the street (N = 1585), sideways to the street (N = 1638), or away from the street resp. pointing to a back-/courtyard (N = 1976). Figure 6 (right panel) shows road traffic noise exposure-response relationships in these groups.

From Figure 6, we found that in the case of road traffic noise, the association between LNight (at the most exposed façade point) and %HSD depended on the orientation of the bedroom towards the nearest street (see also Table S1 in Supplementary File S1). Not surprisingly, the more a bedroom pointed away from the nearest street, the less sleep-disturbed respondents were.

#### 3.3.4. Noise Level Difference between Bedroom Façade and Maximum Façade Point

It has been shown earlier in the literature that having a comparatively “quiet” side in one’s dwelling can reduce annoyance responses to, particularly, road traffic noise exposure [15,23,38]. We were interested if this holds for the outcome %HSD as well. For this, we considered the level difference indicator D_quietside introduced in Section 2.3 “Noise exposure assessment” and examined exposure-response relationships in four groups of D_quietside: (1) less than 5 dB difference, (2) 5−10 dB difference, (3) 10−20 dB difference, (4) more than 20 dB difference. Figure 6 shows the frequency distribution histogram of D_quietside (left panel) and the exposure-response relationships in the four different groups (middle panel). As Figure 6 (middle panel) demonstrates, those with a considerable level difference at their bedroom window to the loudest façade point of the dwelling unit were, expressed in decibels, around 10 to 15 dB “less sleep disturbed” than those with (almost) no level difference. All paired comparisons between the lowest difference category (< 5 dB) and the other difference categories were highly significant (post hoc), despite the difference category variable itself was not significant in the model (cf. Table S1 in Supplementary File S1). However, having the bedroom at the quiet façade does not seem to fully compensate for high noise exposure at the loudest façade point. If this would be the case, we would see shifts in the curves of at least 15 dB between the smallest and the highest difference category (because they also differ by at least 15 dB). But, congruent with expectation, residents clearly seemed to benefit from quiet façades when it comes to their (self-assessed) sleep quality, also at relatively low levels. These results are also well in line with those of the previous section.

#### 3.3.5. Degree of Urbanization

**Figure 7:**
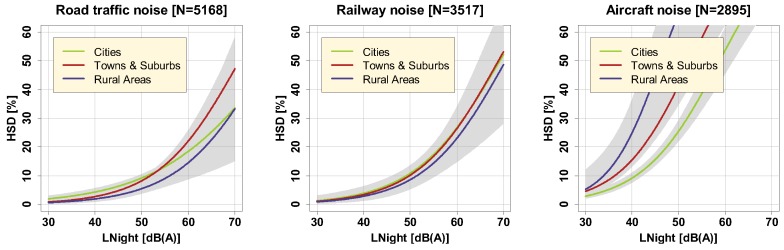
Exposure-response relationships for road traffic (left), railway (middle), and aircraft noise (right), for different degrees of urbanization in the municipality of the respondent’s dwelling unit, including 95% (partly overlapping) confidence intervals (shaded areas). Curves are based on the adjusted model in the full sample with covariates centered on the mean.

Both the overall soundscape at one’s place of living and general lifestyle factors can be assumed to be different between cities, towns/suburbs, and rural areas, and thus the degree of urbanization of one’s dwelling could have an effect on LNight-%HSD relationships. To identify the modifying effect of the degree of urbanization, we employed Eurostat’s DEGURBA indicator [30] which classifies urbanization of the dwelling environment into three categories: cities, towns/suburbs, and rural areas. Exposure-response curves in these three categories are shown in Figure 7.

Tabulated results (cf. Supplementary File S1, Table S1, Section 7) revealed that the effect of the degree of urbanization (DEGURBA) is non-significant for all noise sources. However, we found significant paired differences for %HSD due to aircraft noise between cities and towns/suburbs (−0.537 on the log odds ratio scale, *p* < 0.02, Tukey-adjusted), and cities and rural areas (−0.914 on the log odds ratio scale, *p* < 0.03). According to these observations, aircraft noise is experienced as most sleep-disturbing in rural areas, followed by towns/suburbs, and cities. Maybe masking of aircraft noise in more densely populated areas, with more other noise sources present, is responsible for these differences.

#### 3.3.6. Bedtime and Sleep Duration

**Figure 8:**
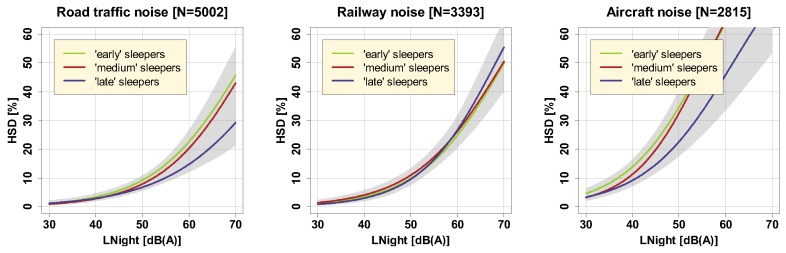
Exposure-response relationships for road traffic (left), railway (middle), and aircraft noise (right), for ‘early’, ‘medium’, and ‘late’ sleepers, including (overlapping) 95% confidence intervals (shaded areas). Curves are based on the adjusted model in the full sample with covariates centered on the mean.

The (potentially) differential effect of both circadian preference and sleep period duration on noise-induced sleep disturbance is acknowledged, but relatively scarcely studied (e.g., in Müller et al. [39]). We tested the effect of circadian preference in three groups of respondents whose estimated sleep onset time was categorized, by tercile split. Results are presented in Figure 8.

Tabulated results (cf. Supplementary File S1, Table S1, Section 8) revealed that the effect of circadian preference is non-significant for all noise sources. By tendency, late sleepers were a bit less disturbed by road traffic and aircraft noise.

**Figure 9:**
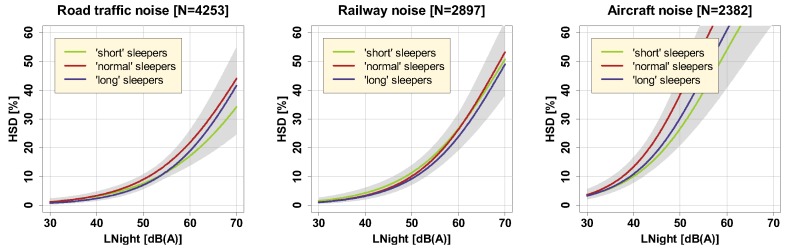
Exposure-response relationships for road traffic (left), railway (middle), and aircraft noise (right), for ‘short’, ‘medium’, and ‘long’ sleepers, including (overlapping) 95% confidence intervals (shaded areas). Curves are based on the adjusted model in the full sample with covariates centered on the mean.

No significant effects (cf. Supplementary File S1, Table S1, Section 9) were found for sleep period duration as well (Figure 9), where, by tercile split, ’short’ sleepers were considered those sleeping less than 7.2 h, ’medium’ sleepers between 7.2 and 8 h, and ’long’ sleepers more than 8 h.

#### 3.3.7. Sleep Medication Intake

**Figure 10:**
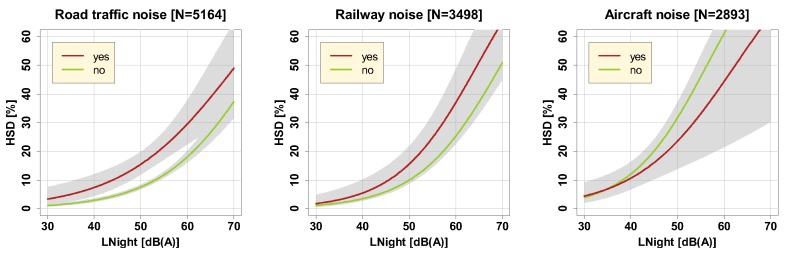
Exposure-response relationships obtained by respondents having declared that they used prescription sleep medication (“yes”) in the four weeks prior to the survey, and by respondents not having taken sleep medication (“no”). Curves are based on the adjusted model in the full sample with covariates centered on the mean and show (partly overlapping) 95% confidence intervals as shaded areas.

Figure 10 shows the effect modification by sleep medication intake. The figure displays exposure-effect curves for those that used sleep medication at least once (N = 371) vs. non-users (N = 4793) in the four weeks before questionnaire completion. While there was no effect in the railway and aircraft noise sample, we found a significant paired difference (−0.837 on the log odds ratio scale, *p* < 0.0001, Tukey-adjusted) between users vs. non-users in the road traffic noise sample, with users having a higher probability to be highly sleep disturbed, corresponding to an Leq-equivalent effect of about 8 dB. The upward shift of the curve of users may indicates that for those who already suffer from road traffic noise-related sleep disturbance, the noise could exacerbate any pre-existing sleep disturbance.

#### 3.3.8. Survey Season (Time of the Year) and average Night Air Temperature

**Figure 11:**
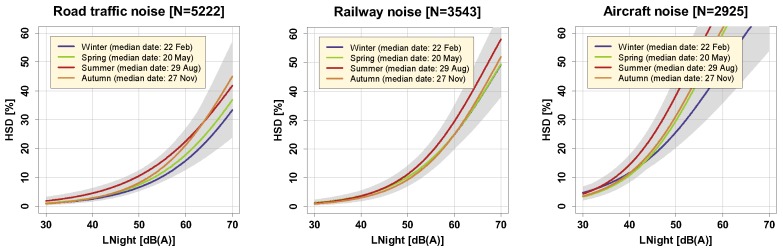
Exposure-response relationships obtained by the responses in the four seasonal waves. “Median date” in the legend refers to the median date of fill-out within the given survey wave. Curves are based on the adjusted model in the full sample with covariates centered on the mean and show (overlapping) 95% confidence intervals as shaded areas.

**Figure 12:**
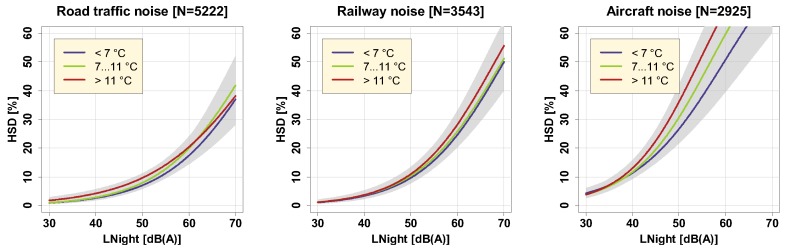
Exposure-response curves for three different night temperature categories including (overlapping) 95% confidence intervals (shaded areas). Curves are based on the adjusted model in the full sample with covariates centered on the mean.

The effect of season (in which a noise survey is carried out) on annoyance responses has been the object of previous studies who all found that annoyance due to noise tends to be higher in the summertime [23,40,41], even if respondents were asked, according to ICBEN’s recommendation [24], to report their average annoyance considering the whole past year. Brink et al. explicitly modeled the effect of day air temperature in the same sample as the present one, and found significant positive associations between temperature and the probability to be highly annoyed by road and railway noise [23]. In the present study, we looked at the effect of both of these factors on P_HSD_ as outcome by stratifying the analyses per survey wave/season (Figure 11), and temperature category (Figure 12), the latter having been obtained by tercile split of the average night temperature in the three months before the survey was carried out.

Despite earlier observations of effect modification by both season and temperature in noise annoyance studies, we could not find similar effects for the outcome P_HSD_, evidenced by non-significant model results (cf. Supplementary File S1, Table S1). This non-difference may be explained by the fact that the average (indoor) noise exposure during time in bed (at night) is not affected by season beyond what is perhaps explainable by seasonally different window positions.

### 3.4. Predictive Power and Goodness-of-Fit of Different Exposure Indicators

Results pertaining to the exercise on predictive power (explained variance) and goodness-of-fit of different exposure metrics with respect to façade point assignment and time windows (see Section 2.4 “Statistical analysis”) are tabulated in Supplementary File S1 in Table S2. Each table section (corresponding to road traffic, railway, and aircraft noise respectively) is sorted by AIC in ascending order to facilitate quick identification of the best fitting exposure indicators.

We observed for road traffic and railway noise that exposure determined at the most exposed façade point yielded higher explained variance (expressed as pseudo R^2^ measures) than assessments at the minimum façade points. At a quick glance, this seems somewhat surprising, since one would assume that bedrooms are rather located on the noise-remote side of buildings and hence the minimum façade point exposure would be a better predictor. At least two tentative explanations are possible for this counterintuitive finding, which are discussed in the next section below. Further, Table S2 in Supplementary File S1 reveals that the estimated bedroom outdoor LNight (only data on road traffic noise were available for this receiver point) performed best to predict %HSD by road traffic noise among all the metrics investigated. This is interesting insofar as one would also expect exposure assessment errors to be on average larger for this measure, which should, for that reason, rather decrease explained variance. Supplementary File S1 contains Figure S1 that shows the three different exposure-response curves for the LNight at the maximum façade point, for the estimated bedroom outdoor LNight, and for the estimated bedroom indoor LNight.

In the case of aircraft noise, the calculated exposure in our study is basically the same at all façade points of a dwelling unit, hence the time period within which the noise exposure was considered becomes the all-determining property. For this source, we found that regressing HSD on LNight (23−07 h) provided the model with the highest explained variance (as evidenced in the pseudo R^2^ measures, cf. Table S2 in Supplementary File S1).

We found that exposure-response models of railway noise yield higher explained variance than road traffic noise models, an observation which echoes findings from an earlier study which revealed that railway noise annoyance was more strongly related to railway noise exposure than road traffic noise annoyance is to road traffic noise exposure [42]. The reasons for this difference are not fully understood. One potential explanation is less exposure misclassification on the side of railway noise. Vienneau et al. [31] argue that the geometry of railways and propagation from this source are relatively simple, as compared to the road network, and thus exposure calculations would become more accurate. Further, the traffic data that fed the exposure calculation model for the railway noise analyses in the present study were more accurate than the data for road traffic noise [26].

## 4. Discussion

### 4.1. Brief Summary

In the present study we established LNight-%HSD relationships that can be used for assessing, nationwide (regarding Switzerland), the public health impacts of road, railway, and aircraft nighttime noise. The fact that adjustment for diverse confounders virtually did not change the exposure-response relationships compared to a crude model with LNight only, is a sign of the robustness of noise exposure as the decisive factor that produces (noise-induced) self-reported sleep disturbance. Given a specific LNight level, the most sleep disturbing noise source was found to be aircraft noise, followed by railway and road traffic noise, which is the same ranking as reported by Basner & McGuire in their meta-analysis carried out for the WHO Environmental Noise Guidelines [13]. We identified noise intermittency, parametrized in the IR indicator [21], to be a relevant factor for the self-assessment of being highly sleep disturbed by road traffic at high and aircraft noise at rather low LNight levels (but not for railway noise). We further found that a bedroom located at the back or the side of buildings, thus increasing noise level difference between loudest and faintest façade point, was associated with less sleep disturbance from road traffic noise, which is in line with previous findings [15,38]. We assume (but could not test the assumption as orientation towards the closest railway tracks was not obtained from respondents) that this would also be the case with railway noise. The LNight-%HSD associations were not significantly affected by bed time and sleep duration, bedroom window position at night, survey season of the year, or outdoor air temperature.

We generally found that noise exposure explained sleep disturbance reactions better if pertaining to the maximum exposed façade point (or, in case of road traffic noise, to the façade point closest to the bedroom), as compared to the minimum exposed (which could only be tested for road traffic and railway noise) – despite that we assumed that most people will, if they can, sleep in a room with a quiet façade. The most straightforward explanation for that is not that the maximum façade point is more “important” in determining %HSD, but that exposure assessment uncertainties are smallest at the maximum façade point. Reducing exposure assessment error in almost all cases strengthens a modeled exposure-response relationship. In urban areas for example, acoustical masking of other community noise sources (i.e., non-traffic) are likely more pronounced at the façades with the minimum noise level, and sound propagation calculations to shielded façades are more challenging. A second explanation could be that exposure at the most exposed façade may generally play a decisive role in the perceived overall noisiness of one’s home, which would lead to both annoyance and self-reported sleep disturbance responses to correlate strongest with the exposure at the maximum façade point.

Despite the fact that for road traffic noise we found the estimated bedroom outdoor level to be a better predictor for %HSD than LNight at the maximum façade point, we considered LNight at the maximum façade point as the relevant exposure measure for all exposure-effect analyses in Section 3.2 “Exposure-response relationships” and “3.3 Effect modification”. Two reasons were responsible for this: First, most noise abatement regulations pertain to the maximum façade point, hence most practical applications of exposure-response curves (e.g., in the scope of defining exposure limits or carrying out environmental noise impact assessments) require exposure values at that façade point, and secondly, concentrating on just one indicator measured at the same façade point for all three sources facilitates comparisons of noise effects between these sources.

### 4.2. Strengths and Limitations

Key advantages of the present study encompass the nation-wide sampling strategy, the use of official register data to draw the survey sample and to eliminate undercoverage, the temporal expansion of the survey in four waves at different times of the year, a fine-grained noise exposure assessment down to the level of 1-h Leq’s at several façade points per dwelling unit, and the assessment of non-response bias, all documented in detail in [23]. A novel aspect of this study is the examination of the roles of effect modification by intermittency ratio (IR), and by other seldom studied effect modifiers.

A potential limitation of the survey was its response rate (31%), which was lower than achieved in previous noise surveys in Switzerland, e.g., [43].

No corrections for multiple statistical testing have been applied. We therefore acknowledge that some of our reported findings that were declared significant, may happened due to chance.

For the entire SiRENE project as a whole, noise exposure for all Swiss buildings and dwelling units was calculated for the reference year 2011. Hence there was a gap between the reference year of noise exposure calculations and the years the survey waves were carried out (2014 and 2015). However, for road traffic and railway noise exposure, slight alterations in local traffic from one year to the next on an established road/rail network have practically no effect on average yearly exposure. However, we acknowledge that changing flight routings, in the present case between 2011 and 2014, could have lead to not fully up-to-date aircraft noise exposure assignments.

A clear drawback of assessing noise-induced sleep disturbance by self-report is the fact that any measure of self-reported sleep disturbance *due to noise* is hardly comparable to similar measures of disturbed sleep when interviewees are asked about their sleep quality in a more general (health survey) framework, i.e., not mentioning noise as the stressor of interest. This has been convincingly demonstrated several decades ago [44], but also recently by Basner & McGuire [13] who in a meta-analysis of noise surveys found that if noise was not mentioned explicitly as the cause of sleep disturbance, the exposure-response relationships become non-significant. These observations could foster the conclusion that survey answers that explicitly refer to noise as the potential cause of sleep disturbance largely exaggerate the true relationship between noise exposure and disturbed sleep. Instead, maybe rather something like attitude to the nighttime noise or to the noise source could be the responsible driver for the increase of self-reported sleep disturbance with LNight. Clearly, objective measures (e.g., EEG awakenings) do not have that problem and henceforth lend themselves way better for comparisons of noise-induced versus other causes of sleep disturbance. However, it is very difficult to obtain larger representative population samples that would undergo the more extensive procedures that are attached to more sophisticated and objective measures of sleep, such as e.g., ambulant polysomnography. But even if these and related methods, e.g., the combination of a single channel ECG with actimetry [45], become more affordable and practical, there is neither expert consensus on a standard method for the assessment of environmental noise effects on sleep, nor is it clear which descriptor(s) of “disturbed sleep” would be the most important one(s) from a public health perspective. Thus, it currently seems that the assessment of the percentage of highly sleep disturbed (%HSD) from self-reports of noise disturbance is the outcome of choice for informing nighttime noise protection policy, evidenced also by its adoption in recent noise-related WHO publications [1,2].

## 5. Conclusions

This study describes noise exposure-response relationships for the outcome “high sleep disturbance” (%HSD) and provides evidence for the effect of noise of different transportation noise sources on self-reported sleep disturbance. The results primarily serve the assessment of road, railway, and aircraft noise impacts on sleep and the setting of source-specific nighttime noise exposure limits. Aircraft noise was found to be a particularly sleep disturbing noise source, followed by railway, and then road traffic noise, reproducing the same order with noise annoyance reactions as found in [23]. We found very clear indications that the bedroom orientation towards the nearest street is a potent effect modifier and thus that sleepers benefit from having their bedrooms at façades shielded from road traffic noise. Builders and architects should therefore consider noise levels at different façade points and assure that apartments always have the option for sleeping in a more quiet room. Given the only small differences in model fit and pseudo R^2^ statistics within the group of Leq-based exposure metrics at the maximum façade point, and in view of the fact that they usually highly correlate, we conclude that as long as noise indicator assessments account for the maximum façade point, they more or less equally well represent the exposure most crucial for inducing sleep disturbance.

## Figures and Tables

**Table 1:** Survey response statistics.

Wave	Survey Period ^a^	Median Fill-Out Date	Postal Returns ^b^ Ge; Fr; It	Online Returns ^b^ Ge; Fr; It	Total Number of Returns	Active Refusals ^c^	Un-delivered ^d^	Response Rate 2 ^e^
1	22 Nov 2014 to 09 Apr 2015	27 Nov 2014	611; 283; 55	255; 74; 9	1287	33	205	29%
2	13 Feb 2015 to 13 May 2015	22 Feb 2015	788; 357; 51	204; 74; 8	1482	30	209	33%
3	12 May 2015 to 12 Aug 2015	20 May 2015	741; 332; 37	227; 69; 10	1416	31	243	31%
4	21 Aug 2015 to 11 Nov 2015	29 Aug 2015	723; 361; 59	179; 71; 14	1407	37	376	31%
Totals:			2863; 1333; 202	865; 288; 41	5592	131	1033	31%

^a^ Dates given are the earliest and latest fill-out dates according to self-report (postal version) or log file (online version); ^b^ Ge = German version; Fr = French version; It = Italian version of questionnaire; ^c^ Questionnaire sent back empty with explanations for not taking part, or addressee called declaring he/she did not want or could not, for any reason, take part in the survey; ^d^ Envelope returned by Swiss post because of invalid/unknown address; ^e^ “Response rate 2” as defined according to the “standard definitions” by the American Association for Public Opinion Research (AAPOR) [32].

**Table 2:** Number of cases (*N*) per LNight category, percent of sample in that category, and observed %HSD in each category. LNight refers to the maximum façade point of the dwelling unit.

	Road			Rail			Air		
LNight (dB(A))	*N*	% of sample	%HSD	*N*	% of sample	%HSD	*N*	% of sample	%HSD
20−25	15	0%	0%	383	11%	1%	577	20%	1%
25−30	93	2%	2%	420	12%	0%	516	18%	2%
30−35	281	5%	1%	466	13%	1%	441	15%	7%
35−40	613	12%	3%	422	12%	2%	445	15%	11%
40−45	916	18%	4%	382	11%	7%	358	12%	15%
45−50	990	19%	6%	368	10%	7%	285	10%	33%
50−55	987	19%	13%	378	11%	15%	222	8%	36%
55−60	867	17%	16%	314	9%	21%	79	3%	39%
60−65	383	7%	21%	259	7%	37%	2	0%	0%
65−70	58	1%	22%	129	4%	33%			
70−75	19	0%	47%	21	1%	57%			
75−80				1	0%	0%			

**Table 3:** Coefficients of logistic regression models for the probability to be highly sleep disturbed (P_HSD_), for road traffic, railway, and aircraft noise LNight at the maximum façade point. The table lists unstandardized (B) and standardized (β) coefficients, standard error of B (SE of B), odds ratios (exp(B) per 1 dB increase), and the variance inflation factor (VIF). Significant *p*-values are highlighted in bold.

**Road traffic noise**							
	**Statistic**	**Intercept**	**LNight (dB(A))**	**Male Sex (vs. Female)**	**Age**	**German** **(vs. FR/IT)**	**Postal Mode (vs. Online)**
**Crude model**	B	−7.2862	0.0978				
	SE of B	0.3495	0.0065				
	exp(B)	0.0007	1.1027				
	β	−2.5727	0.8717				
	VIF	0.0000	0.0000				
	*p*	**<0.01**	**<0.01**				
**Adjusted model**	B	−7.2729	0.0980	−0.0806	0.0036	−0.2712	0.0062
	SE of B	0.4152	0.0065	0.0998	0.0033	0.1002	0.1231
	exp(B)	0.0007	1.1030	0.9226	1.0036	0.7625	1.0063
	β	−2.5470	0.8738	−0.0403	0.0533	−0.1356	0.0031
	VIF	0.0000	1.0090	1.0214	1.0399	1.0058	1.0622
	*p*	**<0.01**	**<0.01**	0.42	0.28	**0.01**	0.96
**Railway noise**							
	**Statistic**	**Intercept**	**LNight (dB(A))**	**Male Sex (vs. Female)**	**Age**	**German** **(vs. FR/IT)**	**Postal Mode (vs. Online)**
**Crude model**	B	−7.7488	0.1121				
	SE of B	0.3428	0.0062				
	exp(B)	0.0004	1.1187				
	β	−3.0417	1.4894				
	VIF	0.0000	0.0000				
	*p*	**<0.01**	**<0.01**				
**Adjusted model**	B	−8.2695	0.1128	0.0133	0.0014	−0.0020	0.5143
	SE of B	0.4389	0.0062	0.1260	0.0042	0.1341	0.1677
	exp(B)	0.0003	1.1195	1.0133	1.0014	0.9980	1.6725
	β	−3.2031	1.4987	0.0066	0.0210	−0.0010	0.2572
	VIF	0.0000	1.0085	1.0211	1.0332	1.0087	1.0591
	*p*	**<0.01**	**<0.01**	0.92	0.74	0.99	**<0.01**
**Aircraft noise**							
	**Statistic**	**Intercept**	**LNight (dB(A))**	**Male Sex (vs. Female)**	**Age**	**German** **(vs. FR/IT)**	**Postal Mode (vs. Online)**
**Crude model**	B	−6.7316	0.1196				
	SE of B	0.3037	0.0069				
	exp(B)	0.0012	1.1271				
	β	−2.5244	1.2058				
	VIF	0.0000	0.0000				
	*p*	<0.01	<0.01				
**Adjusted model**	B	−7.6971	0.1195	0.0396	0.0161	−0.0526	0.2248
	SE of B	0.4274	0.0070	0.1259	0.0043	0.1326	0.1603
	exp(B)	0.0005	1.1270	1.0404	1.0162	0.9487	1.2521
	β	−2.6067	1.2048	0.0198	0.2396	−0.0263	0.1124
	VIF	0.0000	1.0277	1.0223	1.0291	1.0292	1.0496
	*p*	**<0.01**	**<0.01**	0.75	**<0.01**	0.69	0.16

**Table 4:** Coefficients of logistic equations for P_HSD_, as derived for the crude and the adjusted model for each noise source, regressed on the maximum façade point values of (i) Lden, (ii) LNight_22−06 h_ and (iii) LNight_23−07 h._ Covariates in the adjusted models are centered on the mean. To calculate %HSD [%] for a given exposure value x [dB] in the desired noise metric: Insert a, b, and x into the equation %HSD = 100/(1 + exp(−(a + bx))).

		Crude		Adjusted	
Noise Source	Noise Metric	a	b	a	b
Road traffic noise	Lden	−7.2862	0.0978	−8.1988	0.0985
	LNight_22−06 h_	−7.1064	0.0974	−7.1315	0.0976
	LNight_23−07 h_	−7.2862	0.0978	−7.3122	0.0980
Railway noise	Lden	−8.5808	0.1127	−8.6403	0.1135
	LNight_22−06 h_	−7.7105	0.1117	−7.7606	0.1124
	LNight_23−07 h_	−7.7488	0.1121	−7.7996	0.1128
Aircraft noise	Lden	−8.2611	0.1210	−8.3028	0.1212
	LNight_22−06 h_	−6.7154	0.1242	−6.7575	0.1247
	LNight_23−07 h_	−6.7316	0.1196	−6.7649	0.1195

## Supplementary Materials

The following are available online at https://www.mdpi.com/1660-4601/16/21/4186/s1: Supplementary File S1 includes Supplementary Tables S1 and S2, and Supplementary Figure S1, Table S1: Coefficients of binary logistic regression models, Table S2: Explained variance (pseudo R^2^) and goodness-of-fit statistics, Figure S1: %HSD as function of road traffic noise (LNight) in the bedroom, outdoor at the bedroom window, and at the maximum façade point; Supplementary File 2 contains survey questionnaires in German, French, Italian, and English.
