# Supplementary material for: Self-Reported Sleep Disturbance from Road, Rail and Aircraft Noise: Exposure-Response Relationships and Effect Modifiers in the SiRENE Study
Source: Int J Environ Res Public Health. 2019 Oct 29;16(21):4186. doi: 10.3390/ijerph16214186 (PMC6861974; doi:10.3390/ijerph16214186)
Supplement: Supplementary file 1 [file ijerph-16-04186-s001.zip › Supplementary File 2.pdf]

Fragebogen zur SiRENE-Studie

Der vorliegende Fragebogen gliedert sich in die folgenden Teilbereiche: «Angaben zur Person und Wohnsituation»; «Störungen und Belästigungen durch Lärm»; «Zeitnutzung, Schlaf und Schlafqualität»; «Verhaltensweisen und Persönlichkeit»; «Allgemeine Gesundheit und Gesundheitsverhalten»; «Erwerbssituation und Beruf». Die Beantwortung möglichst aller Fragen ist sehr wichtig, auch wenn eine oder mehrere Fragen scheinbar nichts mit Lärm zu tun haben. Vielen Dank!

Bei Fragen mit einem oder mehreren runden Kästchen (❍), kreuzen Sie bitte jeweils die Antwort an, die auf Sie am ehesten zutrifft. Falls mehrere Antworten zutreffen, kreuzen Sie bitte alle zutreffenden Antworten an. Bei Fragen mit einer gestrichelten Linie (..................) oder einem Unterstrich (____) können Sie Wörter oder Zahlen eintragen. Bei Zahlen (1–2–3...) umkreisen Sie einfach die Zahl, die am ehesten zutrifft.

1. Angaben zur Person und zur Wohnsituation

1.1	Heutiges Datum:	1101
1.2	Jahrgang: .... Geschlecht: ❍ Frau ❍ Mann	1201/2
1.3	Ist die Adresse im Begleitbrief der Ort, wo Sie die meiste Zeit wohnen? ❍ ja ❍ nein	1301
1.4	Wie lange wohnen Sie schon in Ihrer jetzigen Wohnung/Haus? .... Jahre	1401
	Wie alt ist das Haus, in dem Sie wohnen? .... Jahre ❍ weiss nicht	1402
1.5	Wohnen Sie: ❍ alleine ❍ als Paar ❍ in einer WG ❍ anderes (❍ mit Kind/ern)	1501
	Wenn Sie sich selbst dazuzählen, wie viele Personen (inkl. Kinder) wohnen insgesamt in Ihrem Haushalt? .... Personen	1502
1.6	In welcher Art Wohnung/Haus wohnen Sie? ❍ Einfamilienhaus / Doppelhaushälfte / Reihenhaus ❍ Wohnung im Erdgeschoss/Parterre ❍ Wohnung im .... Stock	1601<br>1602
	Verfügen Sie in Ihrer Wohnung über... ▪ Schallschutzfenster? ❍ ja ❍ nein ❍ weiss nicht	1603
	▪ Schalldämmlüfter? ❍ ja ❍ nein ❍ weiss nicht	1604
	▪ eine Komfortlüftung? ❍ ja ❍ nein ❍ weiss nicht	1605
	Sind Sie (Mit-)Eigentümer/in oder Mieter/in Ihrer Wohnung oder Ihres Hauses?<br>❍ Hauseigentümer/in ❍ Stockwerkeigentümer/in ❍ Genossenschafter/in ❍ Mieter/in	1606
1.7	Wie ist die Ausrichtung Ihres Schlafzimmers zur nächstgelegenen verkehrsreichen Strasse?<br>❍ von der Strasse weg oder zu einem Innenhof ❍ seitlich zur Strasse ❍ auf die Strasse	1701
	Falls Ihr Schlafzimmer auf die Strasse gerichtet liegt, wie ist der Name dieser Strasse?<br>Name der Strasse:	1702

2. Störungen und Belästigungen durch Lärm

2.1 Wenn Sie an die letzten zwölf Monate bei Ihnen denken, wie stark haben Sie sich durch Lärm von der Strasse, von der Eisenbahn oder von Flugzeugen insgesamt gestört oder belästigt gefühlt?

	überhaupt nicht	etwas	mittelmässig	stark	äusserst	
Strassenlärm	❍	❍	❍	❍	❍	2101
Eisenbahnlärm	❍	❍	❍	❍	❍	2102
Fluglärm	❍	❍	❍	❍	❍	2103

2.2 Wenn Sie an die letzten zwölf Monate bei Ihnen denken, welche Zahl zwischen 0 und 10 gibt am besten an, wie stark Sie sich durch Lärm von der Strasse, von der Eisenbahn oder von Flugzeugen insgesamt gestört oder belästigt fühlten? *(bitte die passende Zahl umkreisen)*

	überhaupt nicht										äusserst	
Strassenlärm	0	1	2	3	4	5	6	7	8	9	10	2201
Eisenbahnlärm	0	1	2	3	4	5	6	7	8	9	10	2202
Fluglärm	0	1	2	3	4	5	6	7	8	9	10	2203

2.3 Werden Sie durch Strassenlärm zu bestimmten Zeiten (am Tag oder in der Nacht) besonders stark gestört oder belästigt?

❍ nein ❍ ja, von _____ : _____ bis _____ : _____ Uhr 2301
und von _____ : _____ bis _____ : _____ Uhr 2302
und von _____ : _____ bis _____ : _____ Uhr 2303

2.4 Werden Sie durch Eisenbahnlärm zu bestimmten Zeiten (am Tag oder in der Nacht) besonders stark gestört oder belästigt?

❍ nein ❍ ja, von _____ : _____ bis _____ : _____ Uhr 2401
und von _____ : _____ bis _____ : _____ Uhr 2402
und von _____ : _____ bis _____ : _____ Uhr 2403

2.5 Werden Sie durch Fluglärm zu bestimmten Zeiten (am Tag oder in der Nacht) besonders stark gestört oder belästigt?

❍ nein ❍ ja, von _____ : _____ bis _____ : _____ Uhr 2501
und von _____ : _____ bis _____ : _____ Uhr 2502
und von _____ : _____ bis _____ : _____ Uhr 2503

2.6 Wenn Sie an die letzten zwölf Monate bei Ihnen denken, wie stark haben Sie sich durch Lärm der folgenden Quellen, in der Nacht, wenn Sie schlafen wollen, gestört oder belästigt gefühlt?

	überhaupt nicht	etwas	mittelmässig	stark	äusserst	
Strassenlärm	❍	❍	❍	❍	❍	2601
Eisenbahnlärm	❍	❍	❍	❍	❍	2602
Fluglärm	❍	❍	❍	❍	❍	2603
Lärm aus der Nachbarschaft	❍	❍	❍	❍	❍	2604
Anderes:	❍	❍	❍	❍	❍	2605/6

2.7 Insgesamt betrachtet: Durch welche Lärmquelle haben Sie sich in Ihrem Wohngebiet in den letzten 12 Monaten am stärksten gestört oder belästigt gefühlt?

❍ Strassenlärm ❍ Lärm von Industrie und Gewerbe 2701
❍ Eisenbahnlärm ❍ Lärm von Nachbarn
❍ Fluglärm ❍ Lärm von Bars/Sport/Freizeitveranstaltungen etc.

❍ anderer Lärm, nämlich: 2702

2.8 Als nächstes geht es um Ihre Empfindlichkeit gegenüber Lärm. Wie stark sind Sie mit folgender Aussage einverstanden?

	ganz und gar einverstanden					gar nicht einverstanden	
▪ Ich bin lärmempfindlich	❍	❍	❍	❍	❍	❍	2801

2.9 Bitte gehen Sie die auf der nächsten Seite folgenden 13 Aussagen der Reihe nach durch. Versuchen Sie bitte, sich in die jeweilige Situation hineinzuversetzen. Kreuzen Sie die Antwortmöglichkeit an, die Ihrer Meinung nach am besten auf Sie zutrifft. → *nächste Seite*

	stimmt genau	stimmt eher	stimmt eher nicht	stimmt gar nicht	
▪ Gesunder Schlaf ist für mich nur in absolut ruhiger Umgebung möglich	❍	❍	❍	❍	2901
▪ Neue Aufgaben kann ich nur in leiser Umgebung bearbeiten	❍	❍	❍	❍	2902
▪ Ich kann mich schnell an Lärm in meiner Wohnumgebung gewöhnen	❍	❍	❍	❍	2903
▪ Ich werde sehr unruhig, wenn ich beim Einschlafen jemanden reden höre	❍	❍	❍	❍	2904
▪ Ich bin sehr empfindlich gegenüber Geräuschen aus meiner Nachbarschaft	❍	❍	❍	❍	2905
▪ Wenn Personen um mich herum laut sind, komme ich mit meiner Arbeit nicht voran	❍	❍	❍	❍	2906
▪ Ich bin geräuschempfindlich	❍	❍	❍	❍	2907
▪ Meine Leistung wird durch eine grosse Geräuschkulisse stark beeinträchtigt	❍	❍	❍	❍	2908
▪ Wenn es nachts laut ist, bin ich morgens unausgeschlafen	❍	❍	❍	❍	2909
▪ Es würde mir nichts ausmachen, an einer lauten Strasse zu wohnen	❍	❍	❍	❍	2910
▪ Für eine ruhige Wohnlage nehme ich andere Nachteile in Kauf	❍	❍	❍	❍	2911
▪ Für anstrengende Arbeiten brauche ich äusserste Ruhe	❍	❍	❍	❍	2912
▪ Ich kann einschlafen, obwohl es laut um mich herum ist	❍	❍	❍	❍	2913

2.10 Im Folgenden geht es um Ihre Einstellung zur Umwelt und zum Lärm. Bitte antworten Sie möglichst spontan.

Was ist Ihnen wichtiger?

❍ Ein starker Wirtschaftsstandort / eine Wirtschaft die sich frei entfalten kann
❍ Ruhe und Umweltschutz / strenge Umwelt-Auflagen — 2100

3. Zeitnutzung, Schlaf und Schlafqualität

3.1 Wann gehen Sie an einem durchschnittlichen Wochentag (Mo-Fr) normalerweise (morgens) aus dem Haus? um _____ : _____ Uhr — 3101

Und wann kehren Sie normalerweise nach Hause zurück? um _____ : _____ Uhr — 3102

3.2 Wie häufig im Monat arbeiten Sie normalerweise in der Nacht, d.h. zwischen 23 Uhr und 6 Uhr morgens?

❍ Nie ❍ Anzahl Nächte pro Monat: .... — 3201

3.3 Die folgenden Fragen betreffen Ihre üblichen Schlafgewohnheiten. Ihre Antworten sollten möglichst genau sein und sich auf die Mehrzahl der Tage und Nächte während der letzten vier Wochen beziehen.

Nach dem Zubettgehen, um welche Uhrzeit etwa löschen Sie normalerweise das Licht?

- während der Woche: (So-Do) um _____ : _____ Uhr — 3301
- am Wochenende: (Fr-Sa) um _____ : _____ Uhr — 3302

und wann stehen Sie normalerweise morgens auf?

- während der Woche: (Mo-Fr) um _____ : _____ Uhr — 3303
- am Wochenende: (Sa-So) um _____ : _____ Uhr — 3304

3.4 Wie häufig passiert es Ihnen, dass Sie...

	nie	selten	manch-mal	häufig	
... schlecht einschlafen	❍	❍	❍	❍	3401
... einen unruhigen Schlaf haben	❍	❍	❍	❍	3402
... nachts mehrmals aufwachen	❍	❍	❍	❍	3403
... morgens zu früh aufwachen	❍	❍	❍	❍	3404
... Sie sich morgens nicht ausgeschlafen fühlen	❍	❍	❍	❍	3405
... Sie wegen Geräuschen im Haus (z.B. vom Partner, Kindern etc.) in Ihrem Schlaf gestört werden oder aufwachen?	❍	❍	❍	❍	3406

3.5 Wie oft haben Sie während der letzten vier Wochen vom Arzt verschriebene (rezeptpflichtige) Schlafmittel eingenommen?

❍ gar nie ❍ weniger als einmal pro Woche
❍ einmal oder zweimal pro Woche ❍ dreimal oder häufiger pro Woche 3501

3.6 In welcher Stellung hielten Sie in den letzten drei Monaten nachts Ihr Schlafzimmerfenster mehrheitlich?

❍ zu ❍ ganz geöffnet ❍ halb geöffnet/angelehnt 3601

Falls das Fenster offen oder halboffen war, warum? ❍ Frischluft ❍ Abkühlung ❍ anderes 3602
Falls das Fenster zu war, warum? ❍ Lärm ❍ Wärmeverlust ❍ anderes 3603

3.7 Wie oft ergreifen Sie zum Schlafen folgende Massnahmen gegen Störungen durch Verkehrslärm (d.h. Strassen-, Eisenbahn-, oder Fluglärm)?

	nie	selten	manch-mal	oft	sehr oft	ständig	
Tragen von Ohrstöpseln/Oropax	❍	❍	❍	❍	❍	❍	3701
Schliessen der Fenster	❍	❍	❍	❍	❍	❍	3702
Änderung der Schlafenszeiten	❍	❍	❍	❍	❍	❍	3703
anderswo schlafen / Zimmer wechseln	❍	❍	❍	❍	❍	❍	3704
Einnahme von Schlaf-/Beruhigungsmitteln	❍	❍	❍	❍	❍	❍	3705
Einnahme von Alkohol	❍	❍	❍	❍	❍	❍	3706
Etwas essen/knabbern	❍	❍	❍	❍	❍	❍	3707
Lesen, Fernsehen, Musik hören, Handy...	❍	❍	❍	❍	❍	❍	3708

4. Verhaltensweisen und Persönlichkeit

4.1 Im Folgenden finden Sie eine Reihe von Feststellungen. Bitte lesen Sie jede Feststellung genau durch und geben Sie an, wie sehr die Aussagen im Allgemeinen auf Sie zutreffen, d.h. wie sehr Ihr übliches Denken und Handeln durch diese Aussagen beschrieben wird.

(bitte die passende Zahl umkreisen)

	stimme nicht zu						stimme völlig zu	
Wenn ich Pläne habe, verfolge ich sie auch	1	2	3	4	5	6	7	4101
Normalerweise schaffe ich alles irgendwie	1	2	3	4	5	6	7	4102
Es ist mir wichtig, an vielen Dingen interessiert zu bleiben	1	2	3	4	5	6	7	4103
Ich kann mehrere Dinge gleichzeitig bewältigen	1	2	3	4	5	6	7	4104
Ich bin entschlossen	1	2	3	4	5	6	7	4105
Ich behalte an vielen Dingen Interesse	1	2	3	4	5	6	7	4106
Ich finde öfter etwas, worüber ich lachen kann	1	2	3	4	5	6	7	4107
Normalerweise kann ich eine Situation aus mehreren Perspektiven betrachten	1	2	3	4	5	6	7	4108
Ich kann mich auch überwinden, Dinge zu tun, die ich eigentlich nicht machen will	1	2	3	4	5	6	7	4109
In mir steckt genügend Energie, um alles zu machen, was ich machen muss	1	2	3	4	5	6	7	4110

4.2 Wie würden Sie Ihre Fähigkeit mit Stress umzugehen auf einer Skala von 1 bis 6 beurteilen? (1 = Ich kann Stress von mir abschütteln, 6 = Stress nagt an mir)

1	2	3	4	5	6	
❍	❍	❍	❍	❍	❍	4201

und wie hoch würden Sie das Ausmass an Stress in Ihrem Leben <u>im letzten Jahr</u> beurteilen (zu Hause und bei der Arbeit)? (1 = keinen Stress, 6 = extremer Stress)

1	2	3	4	5	6	
❍	❍	❍	❍	❍	❍	4202

4.3 Beurteilen Sie bitte, inwiefern die folgenden Aussagen auf Ihr Denken und Handeln in vergangenen <u>unangenehmen oder schwierigen Situationen</u> zutreffen. Bitte machen Sie für jede Aussage eine Angabe.

	**überhaupt nicht**	**ein bisschen**	**ziemlich**	**sehr**	
▪ Ich habe mich mit Arbeit oder anderen Sachen beschäftigt, um auf andere Gedanken zu kommen	❍	❍	❍	❍	4301
▪ Ich habe mich darauf konzentriert, etwas an meiner Situation zu verändern	❍	❍	❍	❍	4302
▪ Ich habe mir eingeredet, dass das alles nicht wahr ist	❍	❍	❍	❍	4303
▪ Ich habe aufmunternde Unterstützung von anderen erhalten	❍	❍	❍	❍	4304
▪ Ich habe es aufgegeben, mich damit zu beschäftigen	❍	❍	❍	❍	4305
▪ Ich habe aktiv gehandelt, um die Situation zu verbessern	❍	❍	❍	❍	4306
▪ Ich wollte einfach nicht glauben, dass mir das passiert	❍	❍	❍	❍	4307
▪ Ich habe meinen Gefühlen freien Lauf gelassen	❍	❍	❍	❍	4308
▪ Ich habe andere Menschen um Hilfe und Rat gebeten	❍	❍	❍	❍	4309
▪ Ich habe versucht, die Dinge von einer positiveren Seite zu betrachten	❍	❍	❍	❍	4310
▪ Ich habe versucht, mir einen Plan zu überlegen, was ich tun kann	❍	❍	❍	❍	4311
▪ Jemand hat mich getröstet und mir Verständnis entgegengebracht	❍	❍	❍	❍	4312
▪ Ich habe gar nicht mehr versucht, die Situation in den Griff zu kriegen	❍	❍	❍	❍	4313
▪ Ich habe versucht, etwas Gutes in dem zu finden, was mir passiert ist	❍	❍	❍	❍	4314
▪ Ich habe etwas unternommen, um mich abzulenken	❍	❍	❍	❍	4315
▪ Ich habe mich damit abgefunden, dass es passiert ist	❍	❍	❍	❍	4316
▪ Ich habe offen gezeigt, wie schlecht ich mich fühle	❍	❍	❍	❍	4317
▪ Ich habe versucht, von anderen Menschen Rat oder Hilfe einzuholen	❍	❍	❍	❍	4318
▪ Ich habe gelernt, damit zu leben	❍	❍	❍	❍	4319
▪ Ich habe mir viele Gedanken darüber gemacht, was hier das Richtige wäre	❍	❍	❍	❍	4320

4.4 Die folgenden Aussagen können mehr oder weniger auf Sie zutreffen. Bitte geben Sie bei jeder Aussage an, inwieweit diese auf Sie persönlich zutrifft.

	**trifft gar nicht zu**	**trifft wenig zu**	**trifft etwas zu**	**trifft ziemlich zu**	**trifft voll und ganz zu**	
▪ Ich habe mein Leben selbst in der Hand	❍	❍	❍	❍	❍	4401
▪ Wenn ich mich anstrenge, werde ich auch Erfolg haben	❍	❍	❍	❍	❍	4402

(Forts.)	trifft gar nicht zu	trifft wenig zu	trifft etwas zu	trifft ziemlich zu	trifft voll und ganz zu	
▪ Egal ob privat oder im Beruf: Mein Leben wird zum grossen Teil von anderen bestimmt	❍	❍	❍	❍	❍	4403
▪ Meine Pläne werden oft vom Schicksal durchkreuzt	❍	❍	❍	❍	❍	4404

5. Gesundheit und Gesundheitsverhalten

5.1 Wie gross sind Sie? .... cm. Wie schwer sind Sie? .... kg. 5101/2

5.2 Wie gut ist Ihr Gesundheitszustand im Allgemeinen?

❍ sehr gut ❍ gut ❍ mittelmässig ❍ schlecht ❍ sehr schlecht 5201

Wurde bei Ihnen jemals durch einen Arzt Diabetes diagnostiziert? ❍ ja ❍ nein 5202

Wurde bei Ihnen jemals durch einen Arzt Bluthochdruck diagnostiziert? ❍ ja ❍ nein 5203

Haben Sie Probleme mit dem Gehör? ❍ ja (→❍ ich trage ein Hörgerät) ❍ nein 5204

5.3 In den folgenden Fragen geht es darum, wie Sie sich fühlen und wie es Ihnen <u>in den vergangenen 4 Wochen</u> gegangen ist. Bitte kreuzen Sie in jeder Zeile die Antwort an, die Ihrem Befinden am ehesten entspricht.

Wie oft waren Sie in den vergangenen 4 Wochen...

	nie	selten	ziemlich oft	meistens	immer	
▪ sehr nervös?	❍	❍	❍	❍	❍	5301
▪ so niedergeschlagen, dass Sie nichts aufheitern konnte?	❍	❍	❍	❍	❍	5302
▪ ruhig und gelassen?	❍	❍	❍	❍	❍	5303
▪ entmutigt und traurig?	❍	❍	❍	❍	❍	5304
▪ glücklich?	❍	❍	❍	❍	❍	5305

5.4 Im folgenden Fragenblock geht es um Aspekte Ihres Gesundheitsverhaltens und um körperliche Aktivitäten.

Rauchen Sie zurzeit (d.h. im letzten Monat) Zigaretten?

❍ Ja (→Ich rauche im Durchschnitt .... Zigaretten pro Tag) 5401/2

❍ Nein (→❍ ich habe früher geraucht, aber schon seit .... Jahren nicht mehr) 5403

❍ Ich habe nie geraucht

Wie viele Stunden und Minuten <u>in der letzten Woche</u> sind Sie in Ihrer Freizeit durch körperliche Betätigung ins Schwitzen gekommen (z.B. durch Rennen/Velofahren usw.)?

___ Stunden und ___ Minuten ***in der letzten Woche*** 5404

Wie lange haben Sie sich <u>in der letzten Woche</u> körperlich so bewegt, dass sie zumindest ein bisschen ausser Atem gekommen sind (z.B. durch zügiges Laufen, Wandern, Tanzen, Gartenarbeiten oder verschiedene Sportarten)?

___ Stunden und ___ Minuten ***in der letzten Woche*** 5405

6. Erwerbssituation und Beruf

6.1 Im Folgenden hätten wir gerne einige Auskünfte über Ihre Erwerbssituation, Ausbildung und Beruf. Sind sie zurzeit erwerbstätig?

❍ ja, Vollzeit ❍ ja, Teilzeit ....% ❍ in Ausbildung (Schule, Studium, Lehre) 6101
❍ nicht erwerbstätig, aber auf Stellensuche ❍ Rentner/in, pensioniert 6102
❍ nicht erwerbstätig und nicht auf Stellensuche (z.B. Hausfrau/Hausmann) ❍ anderes

Falls Sie erwerbstätig sind, welchen Beruf üben Sie im Moment aus? *(möglichst genau)*

.... 6103

6.2 Welchen Beruf hat Ihr Vater bzw. Ihre Mutter ausgeübt als Sie noch ein Kind waren? 6201
Beruf des Vaters: .... Beruf der Mutter: 6202

6.3 Welches ist Ihre höchste abgeschlossene Ausbildung? 6301

❍ Primarschule
❍ Obligatorische Schule: 9 Jahre (Real-, Sekundar-, Bezirksschule etc.)
❍ Übergansausbildungen: Vorlehre, 10. Schuljahr, Berufswahlschule, Haushaltslehrjahr etc.
❍ Diplommittelschule DMS, Fachmittelschule FMS o.ä.
❍ Berufslehre mit Fähigkeitszeugnis/Berufsattest oder Vollzeitberufsschule
❍ Berufs- oder Fach-Maturität
❍ Gymnasiale Maturität, Lehrer/innen-Seminar, Erwachsenen-Maturität
❍ Höhere Berufsbildung mit Fachausweis, Meisterdiplom oder äquivalente Ausbildung
❍ Höhere Fachschule (HF) für Technik, Technikerschule, HF für Wirtschaft (bzw. HKG)
❍ Ingenieurschule HTL, Höhere Fachschule HWV, HFG, HFS o.ä.
❍ Bachelor (Universität, ETH, FH, PH)
❍ Master, Lizentiat, Diplom (Universität, ETH, FH, PH)
❍ Doktorat, PhD, MD

Vielen Dank! Die Befragung ist fast zu Ende. Bitte beantworten Sie noch folgende Fragen:

✎ Möchten Sie an der Verlosung von drei Apple iPad mini Retina teilnehmen? P1
❍ ja ❍ nein

Wären Sie allenfalls daran interessiert, an einer der folgenden wissenschaftlichen Studien als freiwillige (aber bezahlte) Versuchsperson teilzunehmen?
❍ ja, Schlaflabor-Versuch im Zentrum für Chronobiologie an der Universität Basel P2
❍ ja, Lärm-Messungen bei mir zuhause ❍ nein

P3

Dürfen wir Sie ggf. in Zukunft für eine weitere Befragung erneut anfragen? P4
❍ ja ❍ nein

Falls Sie eine oder mehrere der Fragen mit "ja" beantwortet haben, geben Sie uns bitte Ihren Namen sowie Ihre Postadresse, oder Ihre E-Mail-Adresse oder Telefonnummer an, damit wir Sie kontaktieren können:

.... P5

....

☺ Herzlichen Dank für Ihre Mitarbeit! ☺

Fragebogen zur SiRENE-Studie

Rücksendeadresse:

Bundesamt für Umwelt BAFU
Abt. Lärm und NIS
SiRENE-Studie / BMK
3003 Bern

Fax: 058 462 17 57
Email: mark.brink@bafu.admin.ch

Benutzen Sie bitte das adressierte und vorfrankierte Rückantwortcouvert.

Hier ist Platz für Bemerkungen und Kommentare:

Questionnaire de l'étude SiRENE

Le présent questionnaire est subdivisé en six parties : «Informations sur la personne et les conditions d'habitation», «Gêne et perturbations dues au bruit», «Emploi du temps, sommeil et qualité du sommeil», «Comportements et personnalité», «Santé en général et comportement en matière de santé», «Profession et situation professionnelle». Il est important que vous répondiez autant que possible à toutes les questions, même si l'une ou l'autre peut sembler n'avoir rien à faire avec le bruit. D'avance merci !

Pour les questions où vous avez le choix entre plusieurs réponses, veuillez cocher la case (❍) qui correspond le mieux dans votre cas. Si plusieurs réponses sont possibles, veuillez cocher toutes les cases pertinentes. Lorsque vous trouvez une ligne pointillée (..............) ou un soulignement (____), vous pouvez insérer des mots ou des chiffres. Lorsque des chiffres sont proposés (1 2 3...), entourez le chiffre qui convient le mieux.

1. Informations sur la personne et les conditions d'habitation

1.1	Date :	1101
1.2	Année de naissance : .... Sexe : ❍ F ❍ M	1201/2
1.3	L'adresse sur la lettre est-elle votre lieu de résidence principale ? ❍ oui ❍ non	1301
1.4	Depuis combien de temps habitez-vous dans votre appartement/maison actuel ? .... années<br>Quel est l'âge de la maison où vous habitez ? .... annés ❍ ne sais pas	1401<br>1402
1.5	Habitez-vous : ❍ seul/e ❍ en couple ❍ en collocation ❍ autres (❍ avec enfant/s)<br>Combien de personnes au total, vous compris-e (enfants compris) vivent dans votre ménage ? .... personnes	1501<br>1502
1.6	Dans quel type d'appart./maison vivez-vous ? ❍ Maison individiduelle / jumelée / mitoyenne ❍ Appartement au rez-de chaussée ❍ Appartement au .... étage<br>Possédez-vous dans votre appartement...<br>▪ Fenêtres antibruits ? ❍ oui ❍ non ❍ sais pas<br>▪ Aérateurs insonorisés ? ❍ oui ❍ non ❍ sais pas<br>▪ Aération douce ? ❍ oui ❍ non ❍ sais pas<br>Êtes-vous (co)propriétaire ou locataire de votre appartement/maison ?<br>❍ Propriétaire ❍ Copropriétaire ❍ Sociétaire ❍ Locataire	1601<br>1602<br>1603<br>1604<br>1605<br>1606
1.7	Comment votre chambre à coucher est-elle orientée par rapport à la route la plus proche ?<br>❍ à l'opposé de la route ou vers une cour intérieure ❍ latéralement à l'axe routier<br>❍ donne sur la route<br>Si votre chambre à coucher donne sur la route, quel est le nom de celle-ci ?<br>Nom de la route :	1701<br>1702

2. Gêne et perturbations dues au bruit

2.1	Si vous repensez aux douze derniers mois chez vous, à quel point avez-vous été gêné-e ou dérangé-e par le bruit des routes, du chemin de fer ou du trafic aérien? ↻ *tourner svp*	

(suite)

	pas du tout	légè- rement	moyen- nement	forte- ment	extrê- mement	
Bruit routier	❍	❍	❍	❍	❍	2101
Bruit ferroviaire	❍	❍	❍	❍	❍	2102
Bruit du trafic aérien	❍	❍	❍	❍	❍	2103

2.2 Si vous repensez aux douze derniers mois chez vous, quel chiffre entre 0 et 10 exprime le mieux la gêne ou le dérangement que vous a causé globalement le bruit de la route, du chemin de fer ou du trafic aérien ? *(veuillez entourer le chiffre qui convient)*

	0	1	2	3	4	5	6	7	8	9	10	
Bruit routier	0	1	2	3	4	5	6	7	8	9	10	2201
Bruit ferroviaire	0	1	2	3	4	5	6	7	8	9	10	2202
Bruit du trafic aérien	0	1	2	3	4	5	6	7	8	9	10	2203
	pas du tout										extrêmement	

2.3 Le bruit de la route vous gêne-t-il ou vous dérange-t-il spécialement pendant certaines heures (du jour ou de la nuit) ?

❍ non ❍ oui, de _____ h _____ à _____ h _____ 2301
et de _____ h _____ à _____ h _____ 2302
et de _____ h _____ à _____ h _____ 2303

2.4 Le bruit des chemins de fer vous gêne-t-il ou vous dérange-t-il spécialement pendant certaines heures (du jour ou de la nuit) ?

❍ non ❍ oui, de _____ h _____ à _____ h _____ 2401
et de _____ h _____ à _____ h _____ 2402
et de _____ h _____ à _____ h _____ 2403

2.5 Le bruit du trafic aérien vous gêne-t-il ou vous dérange-t-il spécialement pendant certaines heures (du jour ou de la nuit) ?

❍ non ❍ oui, de _____ h _____ à _____ h _____ 2501
et de _____ h _____ à _____ h _____ 2502
et de _____ h _____ à _____ h _____ 2503

2.6 Si vous repensez aux douze derniers mois chez vous, dans quelle mesure le bruit de la route, du chemin de fer ou du trafic aérien vous a-t-il gêné ou dérangé durant la nuit, lorsque vous vouliez dormir ?

	pas du tout	légèrement	moyennement	fortement	extrêmement	
Bruit routier	❍	❍	❍	❍	❍	2601
Bruit ferroviaire	❍	❍	❍	❍	❍	2602
Bruit du trafic aérien	❍	❍	❍	❍	❍	2603
Bruit de voisinage	❍	❍	❍	❍	❍	2604
Autres :	❍	❍	❍	❍	❍	2605/6

2.7 Globalement, quelle source de bruit vous a le plus gêné ou dérangé ces douze derniers mois dans le quartier où vous habitez ?

❍ Bruit routier ❍ Bruit de l'industrie et de l'artisanat
❍ Bruit ferroviaire ❍ Bruit des voisins 2701
❍ Bruit du trafic aérien ❍ Bruit des bars/manifestations sports/loisirs, etc.

❍ autre bruit, à savoir : 2702

2.8	Ici, il s'agit de déterminer votre sensibilité au bruit. Dans quelle mesure êtes-vous d'accord avec l'affirmation suivante ?<br>**tout à fait d'accord** … **pas du tout d'accord**<br>▪ Je suis sensible au bruit ❍ ❍ ❍ ❍ ❍ ❍	2801

2.9	Veuillez répondre aux treize questions qui suivent dans l'ordre. Imaginez-vous dans la situation et cochez la réponse qui vous correspond le mieux.	**tout à fait d'accord**	**plutôt d'accord**	**plutôt pas d'accord**	**pas du tout d'accord**	
	▪ J'ai besoin d'un environnement complètement calme pour avoir une bonne nuit de sommeil	❍	❍	❍	❍	2901
	▪ J'ai besoin d'un environnement calme pour être capable d'effectuer de nouvelles tâches	❍	❍	❍	❍	2902
	▪ Quand je suis à la maison, je m'habitue rapidement au bruit	❍	❍	❍	❍	2903
	▪ Je deviens très agité-e si j'entends quelqu'un parler alors que j'essaie de m'endormir	❍	❍	❍	❍	2904
	▪ Je suis très sensible au bruit de voisinage	❍	❍	❍	❍	2905
	▪ Lorsque les gens autour de moi sont bruyants, j'ai du mal à accomplir mon travail	❍	❍	❍	❍	2906
	▪ Je suis sensible au bruit	❍	❍	❍	❍	2907
	▪ Je suis nettement moins performant-e dans les endroits bruyants	❍	❍	❍	❍	2908
	▪ Je ne me sens pas bien reposé-e lorsque la nuit précédente a été bruyante	❍	❍	❍	❍	2909
	▪ Vivre dans une rue bruyante ne me dérangerait pas	❍	❍	❍	❍	2910
	▪ Je suis prêt-e à accepter certains inconvénients pour vivre dans un endroit calme	❍	❍	❍	❍	2911
	▪ J'ai besoin de calme et de tranquillité pour effectuer un travail difficile	❍	❍	❍	❍	2912
	▪ Je peux m'endormir même quand c'est bruyant	❍	❍	❍	❍	2913

2.10	Cette question concerne votre point de vue au sujet de l'environnement et de l'économie. Veuillez répondre de la façon la plus spontannée possible.<br>Qu'est-ce qui est plus important pour vous ?<br>❍ Une place économique forte / une économie qui peut s'épanouir librement.<br>❍ Le calme et la protection de l'environnement / des normes sévères en matière de protection de l'environnement.	2100

3. Emploi du temps, sommeil et qualité du sommeil

3.1	Quand quittez-vous normalement la maison (le matin) pendant la semaine (lu-ve) ? à _____ h _____	3101
	Et quand retournez-vous normalement à la maison ? à _____ h _____	3102
3.2	Combien de fois par mois travaillez-vous pendant la nuit, c'est-à-dire entre 23 h et 6 h ?<br>❍ Jamais ❍ Nombre de nuits par mois :	3201

3.3 Les questions qui suivent concernent vos habitudes en matière de sommeil. Soyez le plus précis possible dans vos réponses, qui doivent se rapporter à la majorité des jours et des nuits pendant les quatre dernières semaines.

Après vous être couché-e, à quelle heure éteignez-vous normalement la lumière ?

- en semaine (di-je) : à _____ h _____ 3301
- le week-end (ve-sa) : à _____ h _____ 3302

et à quelle heures vous levez-vous d’habitude ?

- en semaine (lu-ve) : à _____ h _____ 3303
- le week-end (sa-di) : à _____ h _____ 3304

3.4 Combien de fois vous arrive-t-il…

	jamais	rarement	parfois	souvent	
... d’éprouver des difficultés à vous endormir	❍	❍	❍	❍	3401
... d’avoir un sommeil agité	❍	❍	❍	❍	3402
... de vous réveiller plusieurs fois au cours de la nuit	❍	❍	❍	❍	3403
... de vous réveiller trop tôt le matin	❍	❍	❍	❍	3404
... de ne pas vous sentir reposé le matin	❍	❍	❍	❍	3405
... d’être dérangé-e ou réveillé-e par des bruits dans la maison (p. ex. partenaire, enfants)	❍	❍	❍	❍	3406

3.5 Combien de fois avez-vous pris des somnifères prescrits par le médecin au cours des quatre dernières semaines ?

❍ jamais ❍ moins d’une fois par semaine
❍ une à deux fois par semaine ❍ trois fois ou plus par semaine 3501

3.6 Dans quelle position se trouvait généralement la fenêtre de votre chambre à coucher pendant la nuit au cours des trois derniers mois ?

❍ fermée ❍ complètement ouverte ❍ à moitié ouverte/en imposte 3601

Si vous aviez la fenêtre ouverte/à moitié ouverte, pourquoi ? ❍ air frais ❍ rafraîssement ❍ autres 3602

Si vous aviez la fenêtre fermée, pourquoi ? ❍ bruit ❍ perte de chaleur ❍ autres 3603

3.7 À quelle fréquence recourez-vous aux mesures ci-dessous pour lutter contre la perturbation de votre sommeil par le bruit du trafic (route, rail, aviation) ?

	jamais	rarement	parfois	souvent	très souvent	toujours	
Bouchons d’oreille/Oropax	❍	❍	❍	❍	❍	❍	3701
Fermer la fenêtre	❍	❍	❍	❍	❍	❍	3702
Modifier les heures de sommeil	❍	❍	❍	❍	❍	❍	3703
Dormir ailleurs / changer de chambre	❍	❍	❍	❍	❍	❍	3704
Prendre des somnifères / calmants	❍	❍	❍	❍	❍	❍	3705
Consommer de l’alcool	❍	❍	❍	❍	❍	❍	3706
Manger / grignoter quelque chose	❍	❍	❍	❍	❍	❍	3707
Lecture, télévision, musique, tél. portable, ...	❍	❍	❍	❍	❍	❍	3708

4. Comportements et personnalité

4.1 Sur une échelle de 1 à 6, comment jugeriez-vous votre capacité à gérer le stress ? (1 = "Je me libère du stress", 6 = "Le stress me ronge")

1	2	3	4	5	6	
❍	❍	❍	❍	❍	❍	4201

Durant l'année dernière, comment jugeriez-vous le niveau de stress dans votre vie (à la maison et au travail) ? (1 = "pas de stress", 6 = "stress extrême")

1	2	3	4	5	6	
❍	❍	❍	❍	❍	❍	4202

4.2 Vous trouvez ci-après un certain nombre d'affirmations. Veuillez les lire attentivement et indiquer dans quelle mesure chacune d'entre elle est vraie d'une manière générale dans votre cas, c'est-à-dire dans quelle mesure elle reflète votre façon de penser et d'agir.

(veuillez entourer le chiffre qui convient)

	**je ne suis pas d'accord**						**je suis entièrement d'accord**	
▪ Quand j'ai des projets, je les mène à bien	1	2	3	4	5	6	7	4101
▪ D'habitude j'arrive à tout faire d'une façon ou d'une autre	1	2	3	4	5	6	7	4102
▪ Pour moi, il est important d'être toujours intéressé-e par beaucoup de choses	1	2	3	4	5	6	7	4103
▪ Je peux faire plusieurs choses en même temps	1	2	3	4	5	6	7	4104
▪ Je suis une personne décidée	1	2	3	4	5	6	7	4105
▪ Je m'intéresse à beaucoup de choses	1	2	3	4	5	6	7	4106
▪ Je trouve souvent des choses qui me font rire	1	2	3	4	5	6	7	4107
▪ Normalement, je peux considérer une situation sous différents angles	1	2	3	4	5	6	7	4108
▪ Je peux me forcer à faire aussi des choses que je n'ai pas vraiment envie de faire	1	2	3	4	5	6	7	4109
▪ J'ai assez d'énergie pour faire tout ce que je dois faire	1	2	3	4	5	6	7	4110

4.3 Nous sommes intéressés par la façon dont les personnes réagissent lorsqu'elles sont confrontées aux évènements difficiles ou stressants dans leur vie. Veuillez indiquer dans quelle mesure les affirmations ci-dessous se sont appliquées à votre façon de penser et d'agir dans des <u>situations passées, désagréables ou difficiles</u>. Merci de donner une réponse pour chaque affirmation.

	**pas du tout**	**un petit peu**	**beau-coup**	**tout à fait**	
▪ Je me suis tourné vers le travail ou d'autres activités pour me changer les idées	❍	❍	❍	❍	4301
▪ J'ai concentré mes efforts pour résoudre la situation	❍	❍	❍	❍	4302
▪ Je me suis dit que ce n'était pas réel	❍	❍	❍	❍	4303
▪ J'ai recherché un soutien émotionnel auprès d'autres	❍	❍	❍	❍	4304
▪ J'ai abandonné l'espoir de faire face	❍	❍	❍	❍	4305
▪ J'ai déterminé une ligne d'action et je l'ai suivie	❍	❍	❍	❍	4306
▪ J'ai refusé de croire que ça m'arrivait	❍	❍	❍	❍	4307
▪ J'ai exprimé mes sentiments négatifs	❍	❍	❍	❍	4308
▪ J'ai essayé d'avoir des conseils ou de l'aide d'autres personnes à propos de ce qu'il fallait faire	❍	❍	❍	❍	4309
▪ J'ai recherché les aspects positifs dans ce qu'il m'arrivait	❍	❍	❍	❍	4310
▪ J'ai planifié les étapes à suivre	❍	❍	❍	❍	4311
▪ J'ai recherché le soutien et la compréhension de quelqu'un	❍	❍	❍	❍	4312
▪ J'ai renoncé à essayer de résoudre la situation	❍	❍	❍	❍	4313
▪ J'ai essayé de voir la situation sous un jour plus positif	❍	❍	❍	❍	4314
▪ J'ai fait quelque chose pour moins y penser	❍	❍	❍	❍	4315
▪ J'ai accepté la réalité de ma nouvelle situation	❍	❍	❍	❍	4316
▪ J'ai évacué mes sentiments déplaisants en en parlant	❍	❍	❍	❍	4317
▪ J'ai recherché l'aide et le conseil d'autres personnes	❍	❍	❍	❍	4318
▪ J'ai appris à vivre dans ma nouvelle situation	❍	❍	❍	❍	4319
▪ J'ai essayé d'élaborer une stratégie à propos de ce qu'il y avait à faire	❍	❍	❍	❍	4320

4.4 Les affirmations ci-dessous peuvent s'appliquer plus ou moins à vous. Veuillez indiquer dans quelle mesure chacune d'entre elles est vraie dans votre cas.

	pas du tout vrai	très peu vrai	un peu vrai	assez vrai	tout à fait vrai	
▪ Je suis maître/maîtresse de ma propre vie	❍	❍	❍	❍	❍	4401
▪ Si je fais des efforts, je peux aussi avoir du succès	❍	❍	❍	❍	❍	4402
▪ Qu'il s'agisse de ma vie privée ou professionnelle, ce sont d'autres surtout qui décident	❍	❍	❍	❍	❍	4403
▪ Le destin contrarie souvent mes projets	❍	❍	❍	❍	❍	4404

5. Santé en général et comportement en matière de santé

5.1 Quelle est votre taille ? .... cm. Quel est votre poids ? .... kg. 5101/2

5.2 Comment est votre état de santé en général ?

❍ très bon ❍ bon ❍ assez bon ❍ mauvais ❍ très mauvais 5201

Un médecin a-t-il jamais diagnostiqué du diabète chez vous ? ❍ oui ❍ non 5202

Un médecin a-t-il jamais diagnostiqué une hypertension chez vous ? ❍ oui ❍ non 5203

Souffrez-vous de problèmes d'ouïe ? ❍ oui (→❍ j'ai un appareil auditif) ❍ non 5204

5.3 Les questions ci-après concernent votre bien-être, comment vous vous êtes senti-e ces <u>quatre dernières semaines</u>. Pour chaque question, veuillez indiquer la réponse qui vous semble la plus appropriée.

Au cours de ces quatre dernières semaines,
y a-t-il eu des moments où...

	jamais	ra-rement	quelques fois	très souvent	en per-manence	
▪ vous vous êtes senti-e très nerveux/nerveuse ?	❍	❍	❍	❍	❍	5301
▪ vous vous êtes senti-e si découragé-e que rien ne pouvait vous remonter le moral ?	❍	❍	❍	❍	❍	5302
▪ vous vous êtes senti-e calme et détendu-e ?	❍	❍	❍	❍	❍	5303
▪ vous vous êtes senti-e triste et abattu-e ?	❍	❍	❍	❍	❍	5304
▪ vous vous êtes senti-e bien dans votre peau ?	❍	❍	❍	❍	❍	5305

5.4 Ce bloc de questions concerne votre comportement en matière de santé et d'exercice physique.

Fumez-vous actuellement (au cours du dernier mois) des cigarettes ?

❍ Oui (→ je fume en moyenne .... cigarettes par jour) 5401/2

❍ Non (→❍ j'ai fumé, mais ne fume plus depuis .... années) 5403

❍ Je n'ai jamais fumé

Pendant combien d'heures et de minutes, au cours de la dernière semaine, avez-vous exercé une activité physique durant vos loisirs qui vous a fait transpirer (p. ex. course à pied, vélo, etc.) ?

_____ heures _____ minutes ***durant la dernière semaine*** 5404

Pendant combien de temps, au cours de la dernière semaine, avez-vous bougé de façon à être au moins un peu essouflé-e (p. ex. marche rapide, randonnée, danse, jardinage ou diverses activités sportives) ?

_____ heures _____ minutes ***durant la dernière semaine*** 5405

6. Profession et situation professionnelle

6.1	Par les questions qui suivent, nous souhaitons réunir quelques informations sur votre situation professionnelle, votre formation et votre profession.<br>Exercez-vous actuellement une activité lucrative ?<br>❍ oui, plein temps ❍ oui, temps partiel ....% ❍ en formation (école, études, apprent.)<br>❍ non, mais recherche un emploi ❍ rentier/rentière, retraité-e<br>❍ non, ne recherche pas d'emploi (p. ex. femme/homme au foyer) ❍ autres<br>Si vous avez une activité lucrative, quel métier exercez-vous actuellement?<br>*(soyez le plus précis possible svp)*<br>..	6101<br>6102<br>6103
6.2	Quel métier exerçait votre père / votre mère lorsque vous étiez enfant ?<br>P. du père : .... P. de la mère :	6201<br>6202
6.3	Quel est le niveau de formation le plus élevé que vous ayez atteint (achevé) ?<br>❍ École primaire<br>❍ École obligatoire : 9 années (enseignement secondaire I)<br>❍ Offres transitoires : préapprentissage, 10e année, école d'orientation professionnelle, année d'apprentissage en intendance, etc.<br>❍ École de degré diplôme (EDD), école de culture générale (ECG) ou analogue<br>❍ Apprentissage avec cert. de capacité/attestation de form. prof. ou école prof. à plein temps<br>❍ Maturité professionnelle ou spécialisée<br>❍ Maturité gymnasiale, école normale, maturité pour adultes<br>❍ Formation professionnelle supérieure avec brevet fédéral, maîtrise fédérale ou formation équivalente<br>❍ École supérieure (ES) en technique, école technique, ES d'économie (ou ESGC)<br>❍ École d'ingénieur ETS, école supérieure spécialisé, ESCEA, ESAA, ESTS ou analogue<br>❍ Bachelor (université, EPF, HES, HEP)<br>❍ Master, licence ou diplôme (université, EPF, HES, HEP)<br>❍ Doctorat	6301

Vous avez presque terminé! Nous vous saurions gré de répondre encore aux questions suivantes :

✎	Souhaitez-vous participer au tirage au sort de trois IPad Mini Retina de la marque Apple ?<br>❍ oui ❍ non	P1
	Seriez-vous intéressé-e à participer à l'une des études scientifiques suivantes comme sujet de recherche volontaire (mais rémunéré) ?<br>❍ oui, essais sur le sommeil en laboratoire, au centre de chronobiologie de l'Université de Bâle<br>❍ oui, mesurages du bruit à mon domicile ❍ non	P2
	Souhaitez-vous être informé-e des résultats de l'étude SiRENE ?<br>❍ oui ❍ non	P3
	Nous autorisez-vous à reprendre éventuellement contact avec vous pour un autre sondage en futur ?<br>❍ oui ❍ non	P4
	Si vous avez répondu par « oui » à une ou plusieurs des questions ci-dessus, nous vous saurions gré de nous indiquer votre nom, votre adresse postale et/ou électronique ou votre numéro de téléphone afin que nous puissions reprendre contact avec vous :<br>..<br>..	P5

☺ Nous vous remercions cordialement de votre participation! ☺

Adresse de retour :

Office Fédérale de l'Environnement OFEV
Div. Bruit et RNI
Étude SiRENE / BMK
3003 Berne

Fax: 058 462 17 57
Courriel: mark.brink@bafu.admin.ch

Veuillez utiliser l’enveloppe affranchie et adressée pour renvoyer le questionnaire.

Vous pouvez utiliser l’espace ci-dessous pour d’éventuels commentaires :

[«SIRPID»]
[2]

Questionario per lo studio SiRENE

Il presente questionario è strutturato nelle seguenti sezioni: «Dati personali e informazioni sulla situazione abitativa», «Effetti nocivi e molesti del rumore», «Impiego del tempo, sonno e qualità del sonno», «Comportamento e personalità», «Stato di salute generale e comportamento in materia di salute», «Situazione lavorativa e professionale». La preghiamo di rispondere a tutte le domande, anche a quelle che apparentemente non hanno nulla a che vedere con il rumore, poiché forniscono indicazioni utili ai fini del sondaggio. Grazie sin da ora per la collaborazione.

Nelle domande in cui figurano una o più caselle tonde (❍) barri la risposta che meglio rispecchia la sua situazione. Nel caso in cui ci siano più risposte che rispecchiano una situazione, barri per favore tutte le risposte corrispondenti. Nelle risposte seguite da una linea tratteggiata (…....) o da una sottolineatura (____) può inserire parole o numeri. Nelle domande seguite da numeri (1-2-3...) metta un cerchio attorno al numero che ritiene più appropriato.

1. Dati personali e informazioni sulla situazione abitativa

1.1	Data odierna (gg.mm.aa):	1101
1.2	Anno di nascita: .... Sesso: ❍ donna ❍ uomo	1201/2
1.3	L'indirizzo sulla lettera di accompagnamento è il suo domicilio abituale? ❍ sì ❍ no	1301
1.4	Da quanti anni abita nell'appartamento / casa attuale? .... anni ❍ non so	1401
	Quanti anni ha la casa in cui vive? .... anni ❍ non so	1402
1.5	Lei vive: ❍ da solo ❍ in coppia ❍ in comunità abitativa ❍ altro (❍ con figli)	1501
	Quante persone (figli compresi) vivono nella sua economia domestica? .... persone	1502
1.6	In che tipo di appartamento / casa vive? ❍ casa unifamiliare / gemella / a schiera ❍ appartamento a pianterreno ❍ appartamento al .... piano	1601<br>1602
	Il suo appartamento è dotato di... ▪ finestre fonoisolanti? ❍ sì ❍ no ❍ non so	1603
	▪ ventilatori insonorizzati? ❍ sì ❍ no ❍ non so	1604
	▪ aerazione controllata? ❍ sì ❍ no ❍ non so	1605
	È (co)proprietario o locatario dell'appartamento o della casa in cui vive?<br>❍ proprietario ❍ comproprietario/a ❍ socio ❍ locatario	1606
1.7	Come è posizionata la sua stanza da letto rispetto alla strada con traffico intenso più vicina?<br>❍ è lontana o dà su un cortile interno ❍ è laterale rispetto alla strada ❍ dà sulla strada	1701
	Se la sua stanza da letto si affaccia sulla strada, indichi il nome della strada.<br>Nome della strada:	1702

2. Effetti nocivi e molesti del rumore

2.1	Ripensando agli ultimi dodici mesi, in che misura si è sentito disturbato o molestato dal rumore del traffico stradale, ferroviario o aereo? ↻ *voltare pagina*	

	per nulla	poco	abbastanza	molto	moltissimo	
Traffico stradale	❍	❍	❍	❍	❍	2101
Traffico ferroviario	❍	❍	❍	❍	❍	2102
Traffico aereo	❍	❍	❍	❍	❍	2103

2.2 Ripensando agli ultimi dodici mesi, da 0 a 10 quanto si è sentito disturbato o molestato dal rumore del traffico stradale, ferroviario o aereo? *(cerchiare il numero che meglio corrisponde alla sua situazione)*

	0	1	2	3	4	5	6	7	8	9	10	
Rumore del Traffico stradale	0	1	2	3	4	5	6	7	8	9	10	2201
Rumore del traffico ferroviario	0	1	2	3	4	5	6	7	8	9	10	2202
Rumore del traffico aereo	0	1	2	3	4	5	6	7	8	9	10	2203
	per nulla										moltissimo	

2.3 Vi sono orari specifici (diurni o notturni) in cui il rumore del traffico stradale la disturba o molesta in maniera particolarmente forte?

❍ no ❍ sì, dalle _____ . _____ alle _____ . _____ 2301
e dalle _____ . _____ alle _____ . _____ 2302
e dalle _____ . _____ alle _____ . _____ 2303

2.4 Vi sono orari specifici (diurni o notturni) in cui il rumore del traffico ferroviario la disturba o molesta in maniera particolarmente forte?

❍ no ❍ sì, dalle _____ . _____ alle _____ . _____ 2401
e dalle _____ . _____ alle _____ . _____ 2402
e dalle _____ . _____ alle _____ . _____ 2403

2.5 Vi sono orari specifici (diurni o notturni) in cui il rumore del traffico aereo la disturba o molesta in maniera particolarmente forte?

❍ no ❍ sì, dalle _____ . _____ alle _____ . _____ 2501
e dalle _____ . _____ alle _____ . _____ 2502
e dalle _____ . _____ alle _____ . _____ 2503

2.6 Ripensando agli ultimi dodici mesi, in che misura si è sentito disturbato o molestato dal rumore di una delle seguenti fonti durante la notte, quando voleva dormire?

	per nulla	poco	abbastanza	molto	moltissimo	
Traffico stradale	❍	❍	❍	❍	❍	2601
Traffico ferroviario	❍	❍	❍	❍	❍	2602
Traffico aereo	❍	❍	❍	❍	❍	2603
Vicinato	❍	❍	❍	❍	❍	2604
Altre fonti:	❍	❍	❍	❍	❍	2605/6

2.7 Da quale fonte di rumore si è sentito in generale maggiormente disturbato o molestato negli ultimi dodici mesi nel luogo in cui abita?

❍ Traffico stradale ❍ Industria e artigianato
❍ Traffico ferroviario ❍ Vicinato 2701
❍ Traffico aereo ❍ Bar/impianti sportivi/manifestazioni, ecc.

❍ Altre fonti di rumore, in particolare: 2702

2.8 Il prossimo punto concerne la sua sensibilità al rumore. È d'accordo con la seguente affermazione?

	**assolutamente d'accordo**					**per nulla d'accordo**	
▪ Sono sensibile ai rumori	❍	❍	❍	❍	❍	❍	2801

2.9 Legga le seguenti 13 affermazioni nell'ordine proposto e cerchi di immedesimarsi nella situazione, apponendo una crocetta sulla risposta che ritiene più appropriata.

	**vero**	**abbast. vero**	**abbast. falso**	**falso**	
▪ Ho bisogno un ambiente assolutamente tranquillo per riuscire a fare una buona notte di sonno	❍	❍	❍	❍	2901
▪ Ho bisogno di silenzio per svolgere nuovi compiti	❍	❍	❍	❍	2902
▪ A casa mi abituo rapidamente al rumore	❍	❍	❍	❍	2903
▪ Mi innervosisco molto se qualcuno parla quando cerco di prendere sonno	❍	❍	❍	❍	2904
▪ Sono molto sensibile ai rumori del vicinato	❍	❍	❍	❍	2905
▪ Non riesco a procedere con il lavoro se intorno a me ci sono persone molto rumorose	❍	❍	❍	❍	2906
▪ Sono sensibile ai rumori	❍	❍	❍	❍	2907
▪ Le mie prestazioni sono molto peggiori quando sono in un ambiente molto rumoroso	❍	❍	❍	❍	2908
▪ Dopo una nottata molto rumorosa non mi sento ben riposato	❍	❍	❍	❍	2909
▪ Non mi infastidirebbe abitare in una strada rumorosa	❍	❍	❍	❍	2910
▪ Pur di abitare in una zona silenziosa accetterei altri svantaggi	❍	❍	❍	❍	2911
▪ Ho bisogno di silenzio assoluto per svolgere lavori impegnativi	❍	❍	❍	❍	2912
▪ Mi addormento facilmente anche quando c'è rumore	❍	❍	❍	❍	2913

2.10 Pensi ora al suo atteggiamento nei confronti dell'ambiente e del rumore, e risponda il più spontaneamente possibile.

Cosa conta di più per lei?

❍ Una piazza economica forte / un libero sviluppo economico

❍ Tranquillità e protezione dell'ambiente / requisiti ambientali severi

2100

3. Impiego del tempo, sonno e qualità del sonno

3.1 Di solito, in un giorno medio (lu-ve) a che ora esce di casa (la mattina)? alle _____ . _____ 3101

E a che ora rincasa solitamente? alle _____ . _____ 3102

3.2 Sull'arco di un mese, quante volte lavora di notte, tra le 23.00 e le 6.00?

❍ Mai ❍ Numero di notti in un mese: 3201

3.3 Nelle ultime quattro settimane quante volte ha assunto sonniferi prescritti dal medico?

❍ mai ❍ meno di una volta a settimana

❍ una o due volte a settimana ❍ tre o più volte a settimana

3501

3.4 Le domande che seguono si riferiscono alle sue abitudini nel dormire. Provi a rispondere nel modo più preciso possibile, secondo le sue abitudini (diurne e notturne) delle ultime quattro settimane.

Di regola, dopo essersi coricato, a che ora circa spegne la luce?

- in settimana (do-gi): alle _____ . _____ 3301
- nel fine settimana (ve-sa): alle _____ . _____ 3302

e a che ora si sveglia il mattino?

- in settimana (lu-ve): alle _____ . _____ 3303
- nel fine settimana (sa-do): alle _____ . _____ 3304

3.4 Quante volte le accade…

	mai	raramente	qualche volta	spesso	
… di addormentarsi male	❍	❍	❍	❍	3401
… di avere un sonno agitato	❍	❍	❍	❍	3402
… di svegliarsi più volte di notte	❍	❍	❍	❍	3403
… di svegliarsi troppo presto al mattino	❍	❍	❍	❍	3404
... non sentirsi riposato al risveglio	❍	❍	❍	❍	3405
... svegliarsi o essere disturbato nel sonno da rumori in casa (p. es. partner, bambini)	❍	❍	❍	❍	3406

3.6 Negli ultimi tre mesi ha dormito con la finestra della stanza da letto prevalentemente?

❍ chiusa ❍ spalancata ❍ semiaperta / socchiusa 3601

Se ha risposto «spalancata» o «semiaperta»,
per quale motivo? ❍ arieggiare ❍ rinfrescare ❍ altro 3602

Se ha risposto «chiusa»,
per quale motivo? ❍ rumore (esterno) ❍ dispersione di calore ❍ altro 3603

3.7 Con che frequenza fa capo ai seguenti provvedimenti per addormentarsi quando non riesce a prendere sonno a causa del rumore causato dal traffico (stradale, ferroviario o aereo)?

	mai	di rado	talvolta	spesso	molto spesso	sempre	
Tappi per le orecchie	❍	❍	❍	❍	❍	❍	3701
Chiudere le finestre	❍	❍	❍	❍	❍	❍	3702
Modificare gli orari del sonno	❍	❍	❍	❍	❍	❍	3703
Dormire altrove / in un'altra stanza	❍	❍	❍	❍	❍	❍	3704
Assunzione di tranquillanti / sonniferi	❍	❍	❍	❍	❍	❍	3705
Bere alcol	❍	❍	❍	❍	❍	❍	3706
Mangiare / sgranocchiare qualcosa	❍	❍	❍	❍	❍	❍	3707
Leggere, guardare la TV, ascoltare musica...	❍	❍	❍	❍	❍	❍	3708

4. Comportamento e personalità

4.1 Legga le affermazioni elencate di seguito e per ognuna indichi in che misura si applica alla sua situazione, vale a dire in che misura rispecchia il suo modo di pensare e agire.

(cerchiare il numero che ritiene più appropriato)

	non corrisponde affatto						corrisponde pienamente	
Quando ho dei progetti, li seguo	1	2	3	4	5	6	7	4101
Di solito riesco sempre nei miei intenti	1	2	3	4	5	6	7	4102
Provare interesse per diverse cose è di grande importanza per me	1	2	3	4	5	6	7	4103
So gestire diverse cose contemporaneamente	1	2	3	4	5	6	7	4104
Sono determinato	1	2	3	4	5	6	7	4105
Ho parecchi interessi	1	2	3	4	5	6	7	4106

	non corrisponde affatto						corrisponde pienamente	
								4107
▪ Trovo spesso qualcosa su cui ridere	1	2	3	4	5	6	7	4108
▪ Di solito riesco a considerare una situazione da tutti i lati	1	2	3	4	5	6	7	4109
▪ Riesco a impormi di fare cose che in realtà non ho voglia di fare	1	2	3	4	5	6	7	4110
▪ Trovo energie sufficienti per fare tutto quello che devo fare	1	2	3	4	5	6	7	

4.2 Utilizzando una scala da 1 a 6, come valuta la sua capacità di gestire lo stress? (1 = "So gestire perfettamente lo stress"; 6 = "Lo stress mi consuma")

1	2	3	4	5	6	
❍	❍	❍	❍	❍	❍	4201

Nel anno passato come valuterebbe la quantità di stress nella sua vita (a casa e sul lavoro)? (1 = "nessuno stress", 6 = "stress molto forte")

1	2	3	4	5	6	
❍	❍	❍	❍	❍	❍	4202

4.3 In che misura le seguenti affermazioni si applicano al suo modo di pensare e agire in <u>situazioni spiacevoli o difficili</u> vissute in passato? Indichi una risposta per affermazione.

	no	un po'	abba- stanza	molto	
▪ Mi sono buttato nel lavoro o in attività varie per pensare ad altro	❍	❍	❍	❍	4301
▪ Mi sono focalizzato su ciò che desidero cambiare della situazione in cui mi trovo	❍	❍	❍	❍	4302
▪ Mi sono autoconvinto che tutto ciò non è vero	❍	❍	❍	❍	4303
▪ Ho ricevuto incoraggiamenti e sostegno da altri	❍	❍	❍	❍	4304
▪ Ho smesso di pensare a questa situazione	❍	❍	❍	❍	4305
▪ Mi sono dato da fare per migliorare la situazione	❍	❍	❍	❍	4306
▪ Non ho voluto credere che ciò possa succedermi	❍	❍	❍	❍	4307
▪ Ho lasciato libero corso ai miei sentimenti	❍	❍	❍	❍	4308
▪ Ho chiesto aiuto e consigli ad altre persone	❍	❍	❍	❍	4309
▪ Ho cercato di vedere il lato positivo delle cose	❍	❍	❍	❍	4310
▪ Ho cercato di pensare cosa fare	❍	❍	❍	❍	4311
▪ Qualcuno mi ha consolato e mostrato comprensione nei miei confronti	❍	❍	❍	❍	4312
▪ Ho rinunciato a gestire la situazione	❍	❍	❍	❍	4313
▪ Ho cercato di ricavare gli aspetti positivi di quanto mi è accaduto	❍	❍	❍	❍	4314
▪ Ho fatto qualcosa per non pensare a questa situazione	❍	❍	❍	❍	4315
▪ Mi sono rassegnato al fatto che ciò è accaduto	❍	❍	❍	❍	4316
▪ Ho mostrato chiaramente il mio malessere	❍	❍	❍	❍	4317
▪ Ho cercato di ottenere aiuto o consigli da altre persone	❍	❍	❍	❍	4318
▪ Ho imparato a convivere con questa situazione	❍	❍	❍	❍	4319
▪ Ho riflettuto a lungo su quale fosse la soluzione migliore	❍	❍	❍	❍	4320

4.4	Le affermazioni seguenti possono essere più o meno pertinenti nel suo caso. Indichi per favore in che misura queste si applicano alla sua situazione personale.						
		**falso**	**piuttosto falso**	**abbast. vero**	**piuttosto vero**	**vero**	
	▪ Ho il controllo della mia vita	❍	❍	❍	❍	❍	4401
	▪ Se mi applico, avrò anch'io successo	❍	❍	❍	❍	❍	4402
	▪ Nella vita privata e nel lavoro: la mia vita è determinata in gran parte dagli altri	❍	❍	❍	❍	❍	4403
	▪ Spesso il destino ci mette lo zampino e manda all'aria i miei progetti	❍	❍	❍	❍	❍	4404

5. Stato di salute generale e comportamento in materia di salute

5.1 La sua altezza: .... cm. Il suo peso: .... kg. 5101/2

5.2 Come va in generale la sua salute?

❍ molto bene ❍ bene ❍ discretamente ❍ male ❍ molto male 5201

Il medico non le ha mai diagnosticato il diabete? ❍ sì ❍ no 5202

Il medico non le ha mai diagnosticato l'ipertensione? ❍ sì ❍ no 5203

Ha problemi di udito? ❍ sì (→ ❍ porto un apparecchio acustico) ❍ no 5204

5.3 Pensi ora al suo stato d'animo e a come si è sentito nelle ultime quattro settimane. Indichi per ogni affermazione la risposta che meglio rispecchia la sua condizione.

Nelle ultime quattro settimane, quante volte le è capitato di sentirsi...

	**mai**	**quasi mai**	**abbast. spesso**	**quasi sempre**	**sempre**	
▪ Molto nervoso?	❍	❍	❍	❍	❍	5301
▪ Così giù di morale che niente avrebbe potuto tirarla su?	❍	❍	❍	❍	❍	5302
▪ Calmo e sereno?	❍	❍	❍	❍	❍	5303
▪ Scoraggiato e triste?	❍	❍	❍	❍	❍	5304
▪ Felice?	❍	❍	❍	❍	❍	5305

5.4 Le domande che seguono riguardano il suo comportamento in materia di salute e l'attività fisica.

Attualmente (riferito all'ultimo mese) fuma sigarette?

❍ sì (→ in media fumo .... sigarette al giorno) 5401/2

❍ no (→❍ ho fumato in passato, ma ho smesso da .... anni) 5403

❍ non ho mai fumato

Nell'ultima settimana, quanto tempo libero ha dedicato a un'attività fisica, arrivando a sudare (p. es. jogging, ciclismo ecc.)?
_____ ore _____ minuti ***nell'ultima settimana*** 5404

Nell'ultima settimana, per quanto tempo ha praticato attività motorie che le hanno causato almeno per poco il fiato corto (p. es. marcia rapida, escursioni, ballare, giardinaggio o sport vari)?
_____ ore _____ minuti ***nell'ultima settimana*** 5405

6. Situazione lavorativa e professionale

6.1	Le chiediamo ora alcune informazioni sulla sua situazione lavorativa, professionale o formativa. Attualmente svolge un'attività lavorativa?<br>❍ sì, a tempo pieno ❍ sì, a tempo parziale ....% ❍ in formazione (scuola, studi, apprendistato)<br>❍ non lavoro, sono in cerca di un impiego ❍ titolare di una rendita, pensionato<br>❍ non lavoro e non sono in cerca di un impiego (p. es. casalinga) ❍ altro<br><br>In caso affermativo: che lavoro svolge attualmente? *(essere il più precisi possibile)*<br>..	6101<br>6102<br>6103
6.2	Che professione hanno svolto sua madre e suo padre quando era bambino?<br>P. del padre: .... P. della madre:	6201<br>6202
6.3	Titolo conseguito (livello più alto):<br>❍ Scuola elementare<br>❍ Scuola dell'obbligo: 9 anni (scuola media, scuola consortile, ecc.)<br>❍ Formazione transitoria: pretirocinio, decimo anno scolastico, ecc.<br>❍ Scuola professionale, scuola specializzata o simile<br>❍ Tirocino professionale con attestato federale di capacità / attestato federale di formazione<br>❍ Maturità professionale o specializzata<br>❍ Maturità liceale, scuola magistrale, maturità per adulti<br>❍ Formazione professionale superiore con attestato professionale federale, diploma di maestria<br>❍ Scuola specializzata superiore di tecnica (SSST), scuola tecnica (ST), scuola superiore di economia aziendale (SSEA) o scuola superiore di informatica di gestione (SSIG)<br>❍ Scuola tecnica superiore (STS), scuola superiore per i quadri dell'economia e dell'amministrazione (SSQEA), scuola superiore di arti applicate (SSAA) o equivalente<br>❍ Bachelor (università, ETH, scuola universitaria professionale, alta scuola pedagogica)<br>❍ Master, licenza o diploma (università, ETH, scuola universitaria professionale, alta scuola pedagogica)<br>❍ Dottorato	6301

Il sondaggio è quasi terminato, la preghiamo di rispondere ancora alle seguenti domande:

✎	Desidera partecipare all'estrazione di tre iPad mini Retina Apple?<br>❍ sì ❍ no	P1
	Sarebbe interessato a partecipare volontariamente (dietro compenso) a uno degli studi scientifici seguenti?<br>❍ sì, a esperimenti nel laboratorio del sonno presso il Centro di cronobiologia a Basilea<br>❍ sì, a misurazioni del rumore presso la mia abitazione ❍ no	P2
	Desidera essere informato in merito ai risultati dello studio SiRENE? ❍ sì ❍ no	P3
	Ci autorizza eventualmente a riprendere contatto con lei in futuro per un'ulteriore sondaggio ? ❍ sì ❍ no	P4
	Se ha risposto «sì» a una o più domande, indichi il suo recapito postale, indirizzo e-mail o numero di telefono al quale possiamo contattarla:<br><br>..<br>..	P5

☺ **La ringraziamo per la sua collaborazione!** ☺

Questionario per lo studio SiRENE

Indirizzo cui inviare il questionario:

Bundesamt für Umwelt BAFU
Abt. Lärm und NIS
SiRENE-Studie / BMK
3003 Bern

Fax: 058 462 17 57
Email: mark.brink@bafu.admin.ch

Utilizzi la busta preaffrancata per rispedire il questionario

Commenti:

["ID"]
[Wave-#]

Questionnaire for the SiRENE study*

** this is not the original questionnaire used for the survey, but the English translation of the original German version and provided for information purposes only. Layout and pagination of this questionnaire may differ from the original German version.*
(c) Federal Office for the Environment, CH-3003 Bern, Switzerland

This questionnaire is divided into the following sub-sections: "Personal information"; "Disturbances and annoyance due to noise"; "Time use, sleep and sleep quality"; "Behavior and personality"; "Health and health behavior"; "Employment situation and occupation". Answering as many questions as possible is very important, even if one or more questions seem to have nothing to do with noise. Thank you very much.

For questions with one or more round boxes, please tick the answer that best applies to you. If multiple answers apply, please tick all applicable answers. For questions with a dashed line (....... ....) or an underscore (____), you can enter words or numbers. For numbers (1 2 3...), simply circle around the number that most applies.

1. Personal information and residential situation

1.1 Today's date: 1101

1.2 Year of birth: .... Sex: ❍ female ❍ male 1201/2

1.3 Is the address in the cover letter the place where you live most of the time? ❍ yes ❍ no 1301

1.4 How long have you been living in your current flat/house? .... years 1401
How old is the house you live in? .... years ❍ don't know 1402

1.5 Do you live: ❍ alone ❍ as a couple ❍ in a flat share ❍ other (❍with children) 1501
Including yourself, how many persons
(incl. children) live in your household? .... persons 1502

1.6 In what kind of apartment / house do you live?
❍ Single-family house / ❍ semi-detached house 1601
❍ row home 1602
❍ Apartment on the ground floor/parterre
❍ Apartment on the ....floor

Does your dwelling have...
- Soundproof windows? ❍ yes ❍ no ❍ don't know 1603
- Sound absorbing fan? ❍ yes ❍ no ❍ don't know 1604
- mechanical ventilation? ❍ yes ❍ no ❍ don't know 1605

Are you (co-)owner or tenant of your apartment or house?
❍ House owner ❍ Apartment owner ❍ Cooperative member ❍ Tenant 1606

1.7 What is the orientation of your bedroom to the nearest busy road/street?
❍ away from the street or to an inner courtyard
❍ sideways to the street ❍ facing the street 1701

If your bedroom is facing the street, what is the name of that street? 1702

Name of street: ....

2. Disturbances and annoyance due to noise

2.1 Thinking about the last twelve months at your home, overall, how much did noise from the road, from railways, or aircraft bother, disturb, or annoy you?

	not at all	slightly	moderately	very	extremely	
Road traffic noise	❍	❍	❍	❍	❍	2101
Railway noise	❍	❍	❍	❍	❍	2102
Aircraft noise	❍	❍	❍	❍	❍	2103

2.2 Thinking about the last twelve month, at your home, what number from 0 to 10 best shows how much you were bothered, disturbed, or annoyed by noise from the road, from railways, or aircraft? *(please circle the appropriate number)*

Road traffic noise 0 1 2 3 4 5 6 7 8 9 10 2201

Railway noise 0 1 2 3 4 5 6 7 8 9 10 2202

Aircraft noise 0 1 2 3 4 5 6 7 8 9 10 2203

Not at all ... Extremely

2.3 Are you particularly disturbed or annoyed by road traffic noise at certain times during the day or night?

❍ no ❍ yes, from _____ : _____ to _____ : _____ o'clock 2301
and from _____ : _____ to _____ : _____ o'clock 2302
and from _____ : _____ to _____ : _____ o'clock 2303

2.4 Are you particularly disturbed or annoyed by railway noise at certain times during the day or night?

❍ no ❍ yes, from _____ : _____ to _____ : _____ o'clock 2401
and from _____ : _____ to _____ : _____ o'clock 2402
and from _____ : _____ to _____ : _____ o'clock 2403

2.4 Are you particularly disturbed or annoyed by aircraft noise at certain times during the day or night?

❍ no ❍ yes, from _____ : _____ to _____ : _____ o'clock 2501
and from _____ : _____ to _____ : _____ o'clock 2502
and from _____ : _____ to _____ : _____ o'clock 2503

2.6 Thinking about the last twelve months at your place, how much did noise from the below sources bother, disturb, or annoy you at night, when you wanted to sleep?

	not at all	slightly	moderately	very	extremely	
Road traffic noise	❍	❍	❍	❍	❍	2601
Railway noise	❍	❍	❍	❍	❍	2602
Aircraft noise	❍	❍	❍	❍	❍	2603
Noise from the neighborhood	❍	❍	❍	❍	❍	2604
Other:	❍	❍	❍	❍	❍	2605/6

2.7 Overall, which noise source have you felt most disturbed or annoyed you in your residential area in the last 12 months?

❍ Road traffic noise ❍ Noise from industry and commerce 2701
❍ Railway noise ❍ Noise from neighbors
❍ Aircraft noise ❍ Noise from bars / sports / recreational events etc.

❍ Other noise, namely: 2702

2.8 Next, it's about your sensitivity to noise. How strongly do you agree with the following statement?

	totally agree					totally disagree	
▪ I am noise sensitive	❍	❍	❍	❍	❍	❍	2801

2.9 Please go through the following 13 statements one by one. Please try to put yourself in the respective situation. Tick the answer that you think best applies to you.

→ *next page*

	strongly agree	agree	disagree	strongly disagree	
▪ I need an absolutely quiet environment to get a good night's sleep	❍	❍	❍	❍	2901
▪ I need quiet surroundings to be able to work on new tasks	❍	❍	❍	❍	2902
▪ When I am at home, I habituate to noise quickly	❍	❍	❍	❍	2903
▪ I become very agitated if I hear someone talking while trying to fall asleep	❍	❍	❍	❍	2904
▪ I am very sensitive to neighborhood noise	❍	❍	❍	❍	2905
▪ When people around me are noisy I don't get on with my work	❍	❍	❍	❍	2906
▪ I'm sensitive to noise	❍	❍	❍	❍	2907
▪ My performance is much worse in noisy places	❍	❍	❍	❍	2908
▪ I do not feel well rested if there has been a lot of noise the night before	❍	❍	❍	❍	2909
▪ It would not bother me to live in a noisy street	❍	❍	❍	❍	2910
▪ For a quiet place to live I would accept other disadvantages	❍	❍	❍	❍	2911
▪ I need peace and quiet to do difficult work	❍	❍	❍	❍	2912
▪ I can fall asleep even when it is noisy	❍	❍	❍	❍	2913

2.10 In the following, it's about your attitude towards the environment and noise. Please answer as spontaneously as possible.

What is more important to you?

❍ A strong business location / an economy that can develop freely
❍ Quietness and environmental protection / strict environmental regulations 2100

3. Time use, sleep and sleep quality

3.1 When do you leave the house (in the morning) on an average weekday (Mon-Fri) at _____ : _____ o'clock 3101

And when do you normally return home? at _____ : _____ o'clock 3102

3.2 How often in the month do you normally work at night, i.e. between 11 p.m. and 6 a.m.? in the morning?

❍ Never ❍ Number of nights per month: 3201

3.3 The following questions concern your usual sleeping habits. Your answers should be as accurate as possible and relate to the majority of days and nights <u>during the last four weeks.</u>

After going to bed, at what time do you normally turn off the lights?

- during the week: (Sun-Thu) at _____ : _____ o'clock 3301
- on the weekend: (Fri-Sat) at _____ : _____ o'clock 3302

and when do you usually get up in the morning?

- during the week: (Mon-Fri) at _____ : _____ o'clock 3303
- on the weekend: (Sat-Sun) at _____ : _____ o'clock 3304

3.4 How often does it happen to you that you...

	never	rarely	sometimes	often	
... fall asleep badly	❍	❍	❍	❍	3401
... have a restless sleep	❍	❍	❍	❍	3402
... wake up several times at night	❍	❍	❍		3403
... wake up too early in the morning	❍	❍	❍	❍	3404
... do not feel well rested in the morning	❍	❍	❍	❍	3405
... are disturbed in your sleep or wake up because of noises in the house (e.g. from your partner, children etc.)?	❍	❍	❍	❍	3406

3.5 How often have you taken prescription sleep medicines during the last four weeks?

❍ never ❍ less than once a week
❍ once or twice a week ❍ three times or more per week 3501

3.6 In which position did you keep your bedroom window during the last three months at night during the majority of the time?

❍ closed ❍ fully open ❍ half open 3601

If the window was open or half-open, why? ❍ fresh air ❍ cooling ❍ other 3602
If the window was closed, why? ❍ noise ❍ heat loss ❍ other 3603

3.7 How often do you take the following measures against noise disturbances (i.e. road, railway or aircraft noise) for sleeping?

	never	rarely	occa-sionally	often	very often	always	
Using earplugs/Oropax	❍	❍	❍	❍	❍	❍	3701
Closing bedroom windows	❍	❍	❍	❍	❍	❍	3702
Change sleeping times	❍	❍	❍	❍	❍	❍	3703
Sleep elsewhere / change rooms	❍	❍	❍	❍	❍	❍	3704
Taking sleeping pills / sedatives	❍	❍	❍	❍	❍	❍	3705
Alcohol intake	❍	❍	❍	❍	❍	❍	3706
Eat or nibble something	❍	❍	❍	❍	❍	❍	3707
Reading, TV, listening to music, smartphone	❍	❍	❍	❍	❍	❍	3708

4. Behavior and personality

4.1 Below you find a number of statements. Please read each statement carefully and indicate how much the statement generally applies to you, i.e. how much your usual thinking and acting is described by these statements.

(please circle the appropriate number)

	disagree						fully agree	
When I have plans, I follow them	1	2	3	4	5	6	7	4101
Normally, I can do anything	1	2	3	4	5	6	7	4102
It's important for me to stay interested in many things	1	2	3	4	5	6	7	4103
I can do several things at once	1	2	3	4	5	6	7	4104
I'm determined	1	2	3	4	5	6	7	4105
I keep an interest in a lot of things	1	2	3	4	5	6	7	4106
I often find something to laugh about	1	2	3	4	5	6	7	4107
Normally, I can well handle a situation from multiple perspectives	1	2	3	4	5	6	7	4108
I can overcome myself to do things that I don't really want to do.	1	2	3	4	5	6	7	4109
I've got enough energy to do anything what I have to do	1	2	3	4	5	6	7	4110

4.2 How would you rate your ability to cope with stress on a scale of 1 to 6?
(1 = I can shake off stress very well, 6 = stress gnaws at me)

1	2	3	4	5	6	
❍	❍	❍	❍	❍	❍	4201

And how high would you rate the level of stress in your life over <u>the past year</u>? (at home and at work)? (1 = no stress, 6 = extreme stress)

1	2	3	4	5	6	
❍	❍	❍	❍	❍	❍	4202

4. Please, rate how much each statement resembles your thoughts and actions in past <u>demanding or difficult situations</u>. Please, endorse each item.

	**not at all**	**a little bit**	**consi-derably**	**very much**	
▪ I've been turning to work or other activities to take my mind off things	❍	❍	❍	❍	4301
▪ I've been concentrating my efforts on doing something about the situation I'm in	❍	❍	❍	❍	4302
▪ I've been saying to myself "this isn't real	❍	❍	❍	❍	4303
▪ I've been getting emotional support from others	❍	❍	❍	❍	4304
▪ I've been giving up trying to deal with it	❍	❍	❍	❍	4305
▪ I've been taking action to try to make the situation better	❍	❍	❍	❍	4306
▪ I've been refusing to believe that it has happened	❍	❍	❍	❍	4307
▪ I've been saying things to let my unpleasant feelings escape	❍	❍	❍	❍	4308
▪ I’ve been getting help and advice from other people	❍	❍	❍	❍	4309
▪ I've been looking for something good in what is happening	❍	❍	❍	❍	4310
▪ I've been trying to come up with a strategy about what to do	❍	❍	❍	❍	4311
▪ I've been getting comfort and understanding from someone	❍	❍	❍	❍	4312
▪ I didn't even try to handle the situation anymore	❍	❍	❍	❍	4313
▪ I've been trying to find something good in this, what happened to me	❍	❍	❍	❍	4314
▪ I did something to distract myself	❍	❍	❍	❍	4315
▪ I've been accepting the reality of the fact that it has happened	❍	❍	❍	❍	4316
▪ I've been expressing my negative feelings	❍	❍	❍	❍	4317
▪ I’ve been trying to get advice or help from other people about what to do	❍	❍	❍	❍	4318
▪ I've been learning to live with it	❍	❍	❍	❍	4319
▪ I've been thinking hard about what steps to take	❍	❍	❍	❍	4320

4.4 The following statements may apply more or less to you. Please indicate the extent to which each statement applies to you personally.

	**totally disagree**				**fully agree**	
▪ I have my life in my own hands	❍	❍	❍	❍	❍	4401
▪ If I make an effort, I will succeed	❍	❍	❍	❍	❍	4402
▪ Whether privately or at work: My life is largely determined by others	❍	❍	❍	❍	❍	4403
▪ My plans are often overshadowed by fate	❍	❍	❍	❍	❍	4404

5. Health and health behavior

5.1	How tall are you? .... cm. What is your weight? ....kg.	5101/2
5.2	How good is your health in general?<br>❍ very good ❍ good ❍ fair ❍ bad ❍ very bad	5201
	Have you ever been diagnosed with diabetes by a doctor? ❍ yes ❍ no	5202
	Have you ever been diagnosed with hypertension by a doctor? ❍ yes ❍ no	5203
	Do you have hearing problems? ❍ yes (❍ I use a hearing aid) ❍ no	5204
5.3	The following questions are about how you feel and how things have been with you <u>during the past 4 weeks</u>. For each question, please give the one answer that comes closest to the way you have been feeling.<br>How much of the time during the past 4 weeks...<br>**none of the time** — **quite sometimes** — **all of the time**	
	▪ have you been a very nervous person? ❍—❍—❍—❍—❍	5301
	▪ have you felt so down in the dumps that nothing could cheer you up? ❍—❍—❍—❍—❍	5302
	▪ have you felt calm and peaceful? ❍—❍—❍—❍—❍	5303
	▪ downhearted and blue? ❍—❍—❍—❍—❍	5304
	▪ have you been a happy person? ❍—❍—❍—❍—❍	5305
5.4	The following block of questions deals with aspects of your health behavior and physical activity.<br>Do you currently (i.e. last month) smoke cigarettes?<br>❍ yes (I smoke on average .... cigarettes per day)<br>❍ no (❍ I used to smoke, but haven't smoked for .... years)<br>❍ I never smoked.	5401/2<br>5403
	How many hours and minutes <u>in the last week</u> did you sweat in your free time due to physical activity (e.g. running, cycling, etc.)?<br>___ hours and ___ minutes in ***the last week***	5404
	How long have you been physically active <u>in the last week</u> so that you have at least lost your breath (e.g. by running, hiking, dancing, gardening or various sports)?<br>___ hours and ___ minutes in ***the last week***	5405

6. Employment situation and occupation

6.1	In the following we would like to have some information about your employment situation.<br>❍ yes, full time ❍ yes, part-time ....% ❍ in training<br>❍ not employed, but looking for a job ❍ retired<br>❍ not employed and not looking for a job (e.g. housewife/houseman) ❍ other	6101<br>6102
	If you are employed, what is your current occupation? *(as accurate as possible)*<br>..	6103
6.2	What profession did your father and mother practice when you were a child?<br>Father's profession: .... Mother 's profession:	6201<br>6202

6.3	What is your highest education?<br>❍ Primary school<br>❍ Compulsory school (9 years)<br>❍ Transition trainings: Pre-apprenticeship, 10th school year, vocational choice school<br>❍ Technical secondary school (DMS, FMS)<br>❍ Apprenticeship with certificate of proficiency/apprenticeship<br>❍ Professional or technical baccalaureate<br>❍ High school diploma, A level ("Matura", "Abitur" in German)<br>❍ Higher vocational education with professional certificate, master's diploma<br>❍ Higher technical college (HF), technical school<br>❍ Engineering School HTL, Higher Vocational School (HWV, HFG, HFS)<br>❍ Bachelor (University, ETH, FH, PH)<br>❍ Master, licentiate, diploma (University, ETH, FH, PH)<br>❍ Doctorate, PhD, MD	6301

Thank you very much. The interview is almost over. Please answer the following questions:

✎	Would you like to take part in the prize draw for three Apple iPad mini Retina?<br>❍ yes ❍ no	P1
	Would you be interested in participating in any of the following scientific studies as a (paid) volunteer?<br>❍ yes, sleep laboratory experiment at the Centre for Chronobiology at the University of Basel<br>❍ yes, noise measurements at my home<br>❍ no	P2<br>P3
	May we ask you again in the future to take part in another survey?<br>❍ yes ❍ no	P4
	If you answered "yes" to one or more of the above questions, please provide us with your name, postal address, e-mail address or telephone number so that we can contact you:<br>..<br>..<br>..	P5

☺ **Thank you very much for your cooperation!** ☺

Return address:

Federal Office for the Environment
Noise and NIR division
SiRENE Study / BMK
3003 Berne

Fax:
email:

Please use the prepaid reply envelope.

Remarks and comments here:
