# Supplementary material for: Self-Reported Sleep Disturbance from Road, Rail and Aircraft Noise: Exposure-Response Relationships and Effect Modifiers in the SiRENE Study
Source: Int J Environ Res Public Health. 2019 Oct 29;16(21):4186. doi: 10.3390/ijerph16214186 (PMC6861974; doi:10.3390/ijerph16214186)
Supplement: Supplementary file 1 [file ijerph-16-04186-s001.zip › Supplementary File 1.pdf]

Supplementary File 1

A) Supplementary Tables

Supplementary Table S1

Coefficients of binary logistic regression models on the probability to be highly sleep disturbed (P_{HSD}), investigating effect modification by stratified categories of IRNight, bedroom window position, bedroom orientation towards street, noise level difference between bedroom façade exposure and loudest (all sources) façade point of the dwelling, degree of urbanization, sleep timing and sleep period duration, sleep medication intake, and season and temperature. "cat." refers to "categories". Significant p-values are highlighted in bold.

Figure # in article	Noise source	Effect modification factor in focus	Effect	B	SE of B	Odds Ratio	p-Value
4 left	Road traffic	IRNight[a]	Intercept	-7.5790	0.4414	0.0005	**<0.01**
			LNight [dB(A)]	0.1032	0.0070	1.1087	**<0.01**
			Male Sex [vs. female]	-0.0811	0.0998	0.9221	0.42
			Age [years]	0.0036	0.0034	1.0036	0.28
			German [vs. FR/IT]	-0.2635	0.1007	0.7683	**<0.01**
			Postal mode [vs. online]	0.0115	0.1232	1.0116	0.93
			IRNight cat. (linear)	-1.5948	0.6885	0.2030	**0.02**
			IRNight cat. (quadratic)	-0.2987	0.6380	0.7418	0.64
			LNight x IRNight cat. (linear)	0.0285	0.0126	1.0289	**0.02**
			LNight x IRNight cat. (quadratic)	0.0039	0.0117	1.0039	0.74
4 center	Railways	IRNight[a]	Intercept	-8.0567	0.5094	0.0003	**<0.01**
			LNight [dB(A)]	0.1092	0.0083	1.1154	**<0.01**
			Male Sex [vs. female]	0.0200	0.1259	1.0202	0.87
			Age [years]	0.0011	0.0042	1.0011	0.80
			German [vs. FR/IT]	-0.0170	0.1346	0.9831	0.90
			Postal mode [vs. online]	0.5071	0.1674	1.6605	**<0.01**
			IRNight cat. (linear)	0.9554	0.6407	2.5997	0.14
			IRNight cat. (quadratic)	0.3300	0.8347	1.3910	0.69
			LNight x IRNight cat. (linear)	-0.0147	0.0129	0.9855	0.25
			LNight x IRNight cat. (quadratic)	-0.0049	0.0158	0.9951	0.76
4 right	Aircraft	IRNight[a]	Intercept	-6.5912	0.4731	0.0014	**<0.01**
			LNight [dB(A)]	0.0971	0.0084	1.1020	**<0.01**
			Male Sex [vs. female]	0.0615	0.1258	1.0634	0.62
			Age [years]	0.0145	0.0043	1.0146	**<0.01**
			German [vs. FR/IT]	-0.0760	0.1336	0.9268	0.57
			Postal mode [vs. online]	0.2228	0.1602	1.2495	0.16
			IRNight cat. (linear)	2.1312	0.5827	8.4252	**<0.01**
			IRNight cat. (quadratic)	-1.7540	0.6524	0.1731	**<0.01**
			LNight x IRNight cat. (linear)	-0.0389	0.0135	0.9618	**<0.01**
			LNight x IRNight cat. (quadratic)	0.0382	0.0150	1.0390	**0.01**
5 left	Road traffic	Window position[b]	Intercept	-7.1144	0.4828	0.0008	**<0.01**
			LNight [dB(A)]	0.0950	0.0080	1.0996	**<0.01**
			Male Sex [vs. female]	-0.0977	0.1010	0.9069	0.33
			Age [years]	0.0037	0.0034	1.0038	0.27
			German [vs. FR/IT]	-0.2690	0.1014	0.7642	**<0.01**

Figure # in article	Noise source	Effect modification factor in focus	Effect	B	SE of B	Odds Ratio	p-Value
			Postal mode [vs. online]	0.0115	0.1243	1.0116	0.93
			Window pos. (linear)	0.8717	0.8315	2.3909	0.29
			Window pos. (quadratic)	-0.6363	0.6351	0.5292	0.32
			LNight x Window pos. (linear)	-0.0155	0.0156	0.9846	0.32
			LNight x Window pos. (quadr.)	0.0138	0.0119	1.0139	0.25
5 middle	Railways	Window position[b]	Intercept	-8.3206	0.5018	0.0002	**<0.01**
			LNight [dB(A)]	0.1122	0.0076	1.1187	**<0.01**
			Male Sex [vs. female]	-0.0165	0.1272	0.9836	0.90
			Age [years]	0.0029	0.0043	1.0029	0.50
			German [vs. FR/IT]	-0.0049	0.1353	0.9951	0.97
			Postal mode [vs. online]	0.5572	0.1695	1.7458	**<0.01**
			Window pos. (linear)	-0.1278	0.7952	0.8800	0.87
			Window pos. (quadratic)	0.6444	0.6529	1.9048	0.32
			LNight x Window pos. (linear)	0.0009	0.0145	1.0009	0.95
			LNight x Window pos. (quadr.)	-0.0066	0.0118	0.9935	0.58
5 right	Aircraft	Window position[b]	Intercept	-7.7066	0.4833	0.0004	**<0.01**
			LNight [dB(A)]	0.1223	0.0087	1.1301	**<0.01**
			Male Sex [vs. female]	0.0422	0.1276	1.0431	0.74
			Age [years]	0.0153	0.0044	1.0154	**<0.01**
			German [vs. FR/IT]	-0.0565	0.1338	0.9451	0.67
			Postal mode [vs. online]	0.2113	0.1618	1.2352	0.19
			Window pos. (linear)	-0.0569	0.7238	0.9446	0.94
			Window pos. (quadratic)	0.1055	0.5617	1.1112	0.85
			LNight x Window pos. (linear)	0.0072	0.0167	1.0072	0.67
6 (middle)	Road traffic	Level difference[c]	Intercept	-7.3400	0.5127	0.0006	**<0.01**
			LNight [dB(A)]	0.0927	0.0087	1.0972	**<0.01**
			Male Sex [vs. female]	-0.0697	0.1013	0.9327	0.49
			Age [years]	0.0038	0.0034	1.0039	0.26
			German [vs. FR/IT]	-0.1818	0.1024	0.8338	0.08
			Postal mode [vs. online]	-0.0064	0.1250	0.9936	0.96
			D_quietside cat. (linear)	-0.3152	0.8607	0.7297	0.71
			D_quietside cat. (quadratic)	1.1853	0.9208	3.2716	0.20
			D_quietside. cat. (cubic)	-0.0169	0.9776	0.9833	0.99
			LNight x D_quietside cat. (linear)	-0.0112	0.0161	0.9889	0.49
			LNight x D_quietside. cat. (quad.)	-0.0169	0.0174	0.9832	0.33
			LNight x D_quietside cat. (cubic)	-0.0031	0.0186	0.9969	0.87
6 (right)	Road traffic	Bedroom orient.[d]	Intercept	-8.2906	0.7884	0.0003	**<0.01**
			LNight [dB(A)]	0.0966	0.0140	1.1014	**<0.01**
			Male Sex [vs. female]	-0.1058	0.1026	0.8996	0.30
			Age [years]	0.0033	0.0034	1.0033	0.34
			German [vs. FR/IT]	-0.2070	0.1033	0.8130	**0.04**
			Postal mode [vs. online]	-0.0246	0.1266	0.9757	0.85
			Bedroom orientation [degree][c]	0.0125	0.0055	1.0126	**0.02**
			LNight x Bedroom orientation	0.0000	0.0001	1.0000	0.65
7 left	Road traffic	DEGURBA[e]	Intercept	-7.5160	0.5720	0.0005	**<0.01**
			LNight [dB(A)]	0.1008	0.0101	1.1060	**<0.01**
			Male Sex [vs. female]	-0.0841	0.1001	0.9193	0.40
			Age [years]	0.0037	0.0034	1.0037	0.27
			German [vs. FR/IT]	-0.2723	0.1011	0.7616	0.01
			Postal mode [vs. online]	0.0120	0.1234	1.0121	0.92
			DEGURBA. (linear)	-1.3455	1.0579	0.2604	0.20
			DEGURBA (quadratic)	0.7305	0.7614	2.0761	0.34
			LNight x DEGURBA (linear)	0.0191	0.0201	1.0193	0.34
			LNight x DEGURBA (quadr.)	-0.0172	0.0144	0.9830	0.23

Figure # in article	Noise source	Effect modification factor in focus	Effect	B	SE of B	Odds Ratio	p-Value
7 middle	Railways	DEGURBA[e]	Intercept	-8.3433	0.5745	0.0002	**<0.01**
			LNight [dB(A)]	0.1131	0.0091	1.1198	**<0.01**
			Male Sex [vs. female]	0.0230	0.1265	1.0232	0.86
			Age [years]	0.0017	0.0042	1.0017	0.68
			German [vs. FR/IT]	-0.0200	0.1381	0.9802	0.89
			Postal mode [vs. online]	0.5109	0.1681	1.6667	**<0.01**
			DEGURBA (linear)	-0.3835	1.0116	0.6815	0.70
			DEGURBA (quadratic)	0.0635	0.7123	1.0656	0.93
			LNight x DEGURBA (linear)	0.0041	0.0183	1.0041	0.82
			LNight x DEGURBA (quadr.)	-0.0022	0.0128	0.9978	0.86
7 right	Aircraft	DEGURBA[e]	Intercept	-8.3845	0.6460	0.0002	**<0.01**
			LNight [dB(A)]	0.1450	0.0141	1.1560	**<0.01**
			Male Sex [vs. female]	0.0575	0.1279	1.0591	0.65
			Age [years]	0.0157	0.0043	1.0158	**<0.01**
			German [vs. FR/IT]	-0.0579	0.1389	0.9437	0.68
			Postal mode [vs. online]	0.1936	0.1618	1.2136	0.23
			DEGURBA (linear)	-0.7696	1.1538	0.4632	0.50
			DEGURBA (quadratic)	-0.5207	0.7902	0.5941	0.51
			LNight x DEGURBA (linear)	0.0402	0.0284	1.0410	0.16
			LNight x DEGURBA (quadr.)	0.0129	0.0192	1.0130	0.50
8 left	Road traffic	Sleep timing[f]	Intercept	-7.2930	0.4298	0.0007	**<0.01**
			LNight [dB(A)]	0.1000	0.0068	1.1052	**<0.01**
			Male Sex [vs. female]	-0.0518	0.1039	0.9495	0.62
			Age [years]	0.0019	0.0035	1.0019	0.59
			German [vs. FR/IT]	-0.2750	0.1039	0.7595	**<0.01**
			Postal mode [vs. online]	-0.0483	0.1249	0.9528	0.70
			Sleep timing cat. (linear)	0.4240	0.6293	1.5281	0.50
			Sleep timing cat. (quadratic)	0.5057	0.6473	1.6581	0.43
			LNight x Sleep timing cat. (lin.)	-0.0132	0.0116	0.9869	0.25
			LNight x Sleep timing cat. (q.)	-0.0101	0.0119	0.9899	0.40
8 middle	Railways	Sleep timing[f]	Intercept	-8.1787	0.4534	0.0003	**<0.01**
			LNight [dB(A)]	0.1127	0.0064	1.1193	**<0.01**
			Male Sex [vs. female]	-0.0411	0.1312	0.9598	0.75
			Age [years]	0.0002	0.0044	1.0002	0.96
			German [vs. FR/IT]	0.0004	0.1385	1.0004	1.00
			Postal mode [vs. online]	0.4898	0.1690	1.6320	**<0.01**
			Sleep timing cat. (linear)	-0.4608	0.6350	0.6308	0.47
			Sleep timing cat. (quadratic)	-0.5587	0.5992	0.5720	0.35
			LNight x Sleep timing cat. (lin.)	0.0089	0.0113	1.0089	0.43
			LNight x Sleep timing cat. (q.)	0.0090	0.0109	1.0090	0.41
8 right	Aircraft	Sleep timing[f]	Intercept	-7.5692	0.4433	0.0005	**<0.01**
			LNight [dB(A)]	0.1194	0.0074	1.1268	**<0.01**
			Male Sex [vs. female]	0.0714	0.1320	1.0740	0.59
			Age [years]	0.0144	0.0044	1.0145	**<0.01**
			German [vs. FR/IT]	-0.1497	0.1370	0.8610	0.27
			Postal mode [vs. online]	0.1632	0.1627	1.1772	0.32
			Sleep timing cat. (linear)	0.0359	0.5416	1.0366	0.95
			Sleep timing cat. (quadratic)	0.6687	0.5908	1.9516	0.26
			LNight x Sleep timing cat. (lin.)	-0.0091	0.0123	0.9909	0.46
			LNight x Sleep timing cat. (q.)	-0.0167	0.0132	0.9835	0.21
9 left	Road traffic	Sleep duration[g]	Intercept	-7.5085	0.4667	0.0005	**<0.01**
			LNight [dB(A)]	0.1023	0.0074	1.1077	**<0.01**
			Male Sex [vs. female]	-0.0891	0.1140	0.9147	0.43
			Age [years]	0.0044	0.0038	1.0045	0.24

Figure # in article	Noise source	Effect modification factor in focus	Effect	B	SE of B	Odds Ratio	p-Value
			German [vs. FR/IT]	-0.3212	0.1129	0.7252	**<0.01**
			Postal mode [vs. online]	-0.0277	0.1287	0.9726	0.83
			Sleep duration cat. (linear)	-0.7639	0.7067	0.4659	0.28
			Sleep duration cat. (quadratic)	-0.1070	0.6796	0.8985	0.87
			LNight x Sleep duration cat. (lin.)	0.0141	0.0129	1.0142	0.28
			LNight x Sleep duration cat. (q.)	-0.0015	0.0125	0.9985	0.91
9 middle	Railways	Sleep duration[g]	Intercept	-7.9778	0.4783	0.0003	**<0.01**
			LNight [dB(A)]	0.1104	0.0068	1.1167	**<0.01**
			Male Sex [vs. female]	-0.0532	0.1435	0.9481	0.71
			Age [years]	-0.0011	0.0046	0.9989	0.81
			German [vs. FR/IT]	-0.0237	0.1493	0.9766	0.87
			Postal mode [vs. online]	0.5283	0.1724	1.6961	**<0.01**
			Sleep duration cat. (linear)	-0.4403	0.6400	0.6438	0.49
			Sleep duration cat. (quadratic)	0.3124	0.6765	1.3667	0.64
			LNight x Sleep duration cat. (lin.)	0.0056	0.0115	1.0056	0.63
			LNight x Sleep duration cat. (q.)	-0.0060	0.0121	0.9940	0.62
9 right	Aircraft	Sleep duration[g]	Intercept	-8.0869	0.4929	0.0003	**<0.01**
			LNight [dB(A)]	0.1277	0.0082	1.1362	**<0.01**
			Male Sex [vs. female]	0.0809	0.1459	1.0842	0.58
			Age [years]	0.0160	0.0049	1.0162	**<0.01**
			German [vs. FR/IT]	-0.0412	0.1516	0.9596	0.79
			Postal mode [vs. online]	0.2203	0.1700	1.2465	0.19
			Sleep duration cat. (linear)	-0.2269	0.6255	0.7970	0.72
			Sleep duration cat. (quadratic)	0.3438	0.6246	1.4103	0.58
			LNight x Sleep duration cat. (lin.)	0.0071	0.0140	1.0071	0.61
			LNight x Sleep duration cat. (q.)	-0.0143	0.0141	0.9858	0.31
10 left	Road traffic	Sleep medic.	Intercept	-7.3763	0.4443	0.0006	<0.01
			LNight	0.0993	0.0071	1.1044	**<0.01**
			Male Sex [vs. female]	-0.0469	0.1017	0.9542	0.64
			Age	0.0020	0.0034	1.0020	0.55
			German [vs. FR/IT]	-0.2208	0.1023	0.8019	0.03
			Postal mode [vs. online]	-0.0337	0.1241	0.9669	0.79
			Sleep medication intake [vs. not]	1.6308	1.0288	5.1082	0.11
			LNight x Sleep medication intake	-0.0165	0.0191	0.9837	0.39
10 middle	Railways	Sleep medic.	Intercept	-8.2086	0.4532	0.0003	**<0.01**
			LNight	0.1118	0.0065	1.1183	**<0.01**
			Male Sex [vs. female]	0.0287	0.1275	1.0292	0.82
			Age	-0.0002	0.0043	0.9998	0.97
			German [vs. FR/IT]	0.0394	0.1360	1.0402	0.77
			Postal mode [vs. online]	0.4991	0.1681	1.6472	**<0.01**
			Sleep medication intake [vs. not]	0.3161	1.1827	1.3718	0.79
			LNight x Sleep medication intake	0.0039	0.0218	1.0039	0.86
10 right	Aircraft	Sleep medic.	Intercept	-7.7647	0.4422	0.0004	**<0.01**
			LNight	0.1216	0.0074	1.1293	**<0.01**
			Male Sex [vs. female]	0.0397	0.1273	1.0405	0.75
			Age	0.0162	0.0043	1.0163	**<0.01**
			German [vs. FR/IT]	-0.0718	0.1338	0.9308	0.59
			Postal mode [vs. online]	0.2193	0.1609	1.2452	0.17
			Sleep medication intake [vs. not]	0.9243	1.1884	2.5202	0.44
			LNight x Sleep medication intake	-0.0267	0.0277	0.9737	0.34
11 left	Road traffic	Season[h]	Intercept	-7.4952	0.7559	0.0006	**<0.01**
			LNight [dB(A)]	0.0976	0.0133	1.1026	**<0.01**
			Male Sex [vs. female]	-0.0775	0.1000	0.9254	0.44
			Age [years]	0.0034	0.0033	1.0034	0.31

Figure # in article	Noise source	Effect modification factor in focus	Effect	B	SE of B	Odds Ratio	p-Value
			German [vs. FR/IT]	-0.2555	0.1006	0.7745	**0.01**
			Postal mode [vs. online]	0.0135	0.1238	1.0136	0.91
			Spring (vs. Winter)	0.1014	1.0285	1.1067	0.92
			Summer (vs. Winter)	0.8936	0.9613	2.4439	0.35
			Autumn (vs. Winter)	-0.4195	1.0576	0.6574	0.69
			LNight x Spring vs. Winter	0.0008	0.0189	1.0008	0.97
			LNight x Summer vs. Winter	-0.0076	0.0178	0.9924	0.67
			LNight x Autumn vs. Winter	0.0130	0.0194	1.0131	0.50
11 middle	Railways	Season[h]	Intercept	-7.9677	0.7170	0.0003	**<0.01**
			LNight [dB(A)]	0.1068	0.0119	1.1127	**<0.01**
			Male Sex [vs. female]	0.0111	0.1263	1.0112	0.93
			Age [years]	0.0013	0.0042	1.0013	0.76
			German [vs. FR/IT]	0.0002	0.1344	1.0002	1.00
			Postal mode [vs. online]	0.5083	0.1685	1.6625	**<0.01**
			Spring (vs. Winter)	-0.0193	0.9396	0.9809	0.98
			Summer (vs. Winter)	-0.5253	0.9460	0.5914	0.58
			Autumn (vs. Winter)	-0.6299	1.0050	0.5326	0.53
			LNight x Spring vs. Winter	0.0004	0.0171	1.0004	0.98
			LNight x Summer vs. Winter	0.0126	0.0170	1.0126	0.46
			LNight x Autumn vs. Winter	0.0105	0.0180	1.0106	0.56
11 right	Aircraft	Season[h]	Intercept	-6.8414	0.6297	0.0011	**<0.01**
			LNight [dB(A)]	0.0970	0.0129	1.1019	**<0.01**
			Male Sex [vs. female]	0.0382	0.1266	1.0389	0.76
			Age [years]	0.0161	0.0043	1.0162	**<0.01**
			German [vs. FR/IT]	-0.0416	0.1333	0.9593	0.76
			Postal mode [vs. online]	0.2162	0.1613	1.2414	0.18
			Spring (vs. Winter)	-1.2146	0.8547	0.2968	0.16
			Summer (vs. Winter)	-1.1207	0.8197	0.3261	0.17
			Autumn (vs. Winter)	-1.1605	0.8775	0.3133	0.19
			LNight x Spring vs. Winter	0.0282	0.0194	1.0287	0.14
			LNight x Summer vs. Winter	0.0342	0.0186	1.0348	0.07
			LNight x Autumn vs. Winter	0.0288	0.0200	1.0292	0.15
12 left	Road traffic	Temperature[i]	Intercept	-7.3438	0.4182	0.0006	**<0.01**
			LNight [dB(A)]	0.0986	0.0066	1.1037	**<0.01**
			Male Sex [vs. female]	-0.0757	0.0999	0.9271	0.45
			Age [years]	0.0035	0.0033	1.0035	0.30
			German [vs. FR/IT]	-0.2285	0.1024	0.7957	**0.03**
			Postal mode [vs. online]	0.0135	0.1240	1.0136	0.91
			Temperature cat. (linear)	0.7597	0.6031	2.1377	0.21
			Temperature cat. (quadratic)	0.4873	0.6285	1.6279	0.44
			LNight x Temperature cat. (linear)	-0.0104	0.0111	0.9897	0.35
			LNight x Temperature cat. (q.)	-0.0090	0.0116	0.9910	0.43
12 middle	Railways	Temperature[i]	Intercept	-8.2718	0.4394	0.0003	**<0.01**
			LNight [dB(A)]	0.1125	0.0062	1.1191	**<0.01**
			Male Sex [vs. female]	0.0138	0.1261	1.0139	0.91
			Age [years]	0.0014	0.0042	1.0014	0.75
			German [vs. FR/IT]	0.0252	0.1373	1.0256	0.85
			Postal mode [vs. online]	0.5136	0.1687	1.6713	**<0.01**
			Temperature cat. (linear)	-0.0420	0.6003	0.9589	0.94
			Temperature cat. (quadratic)	-0.1522	0.5936	0.8588	0.80
			LNight x Temperature cat. (linear)	0.0029	0.0108	1.0029	0.79
			LNight x Temperature cat. (q.)	0.0029	0.0107	1.0029	0.78
12 right	Aircraft	Temperature[i]	Intercept	-7.7071	0.4282	0.0004	**<0.01**
			LNight [dB(A)]	0.1193	0.0071	1.1267	**<0.01**

Figure # in article	Noise source	Effect modification factor in focus	Effect	B	SE of B	Odds Ratio	p-Value
			Male Sex [vs. female]	0.0320	0.1263	1.0325	0.80
			Age [years]	0.0159	0.0043	1.0161	**<0.01**
			German [vs. FR/IT]	-0.0106	0.1355	0.9895	0.94
			Postal mode [vs. online]	0.2215	0.1609	1.2480	0.17
			Temperature cat. (linear)	-0.6986	0.5307	0.4973	0.19
			Temperature cat. (quadratic)	0.1079	0.5355	1.1139	0.84
			LNight x Temperature cat. (linear)	0.0202	0.0120	1.0204	0.09
			LNight x Temperature cat. (q.)	-0.0018	0.0122	0.9982	0.88

[a] Modeled as ordered factor with three levels, thus results consider linear and quadratic contrasts; Coding: value 1 for "low", 2 for "medium", and 3 for "high" IR

[b] Modeled as ordered factor with three levels, thus results consider linear and quadratic contrasts; Coding: value 0 for "closed", 1 for "half open/tilted", 2 for "fully open" windows

[c] Modeled as ordered factor with four levels, thus results consider linear, quadratic, and cubic contrasts; Coding: value 1 for <5 dB category, 2 for 5-10 dB category, 3 for 10-20 dB category, 4 for >20 dB category

[d] Assumed an interval scaled predictor with the values 0 (away from street, corresponding to an angle of 0°), 90 (sideways to street, angle 90°), 180 (pointing towards street, angle 180°)

[e] Degree of urbanization indicator used by Eurostat, modeled as ordered factor with three levels, thus results consider linear and quadratic contrasts; Coding: value 1 for "cities", 2 for "towns and suburbs", and 3 for "rural areas"

[f] Modeled as ordered factor with three levels, thus results consider linear and quadratic contrasts; Coding: value 1 for "early", 2 for "medium", 3 for "late" sleepers

[g] Modeled as ordered factor with three levels, thus results consider linear and quadratic contrasts; Coding: value 1 for "short", 2 for "medium", 3 for "long" sleepers

[h] Reference category: Winter

[i] Modeled as ordered factor with three levels, thus results consider linear and quadratic contrasts; Coding: value 1 for < 7 °C category, value 2 for 7 - 11 °C category, value 3 for > 11 °C category

Supplementary Table S2

Explained variance (pseudo R^2) and goodness-of-fit statistics of adjusted models using different noise exposure indicators (regarding façade point assignments and time windows) on which the probability of being highly sleep disturbed (P_{HSD}) was regressed, with covariates centered on the mean. Abbreviations: AIC=Akaike Information Criterion; BIC=Bayesian Information Criterion; M-Z R^2=McKelvey&Zavoina pseudo R^2; NK R^2=Nagelkerke pseudo R^2. The tables for each noise source are sorted in ascending order of AIC.

Road traffic noise:

Exposure indicator	AIC	BIC	M-Z R^2	NK R^2	Log-Likelihood	Deviance
Estimated bedroom outdoor LNight	2820.16	2859.52	0.26	0.16	-1404.08	2808.16
Leq05-06h at max. façade	2941.79	2981.16	0.20	0.11	-1464.9	2929.79
Leq06-07h at max. façade	2942.45	2981.82	0.20	0.11	-1465.23	2930.45
LNight at max. façade	2942.52	2981.88	0.20	0.11	-1465.26	2930.52
Lden at max. façade	2942.57	2981.94	0.20	0.11	-1465.29	2930.57
Leq24 at max. façade	2942.62	2981.98	0.20	0.11	-1465.31	2930.62
LDay at max. façade	2942.64	2982.00	0.20	0.11	-1465.32	2930.64
Leq01-05h at max. façade	2942.89	2982.25	0.20	0.11	-1465.44	2930.89
Leq19-23h at max. façade	2943.52	2982.88	0.20	0.11	-1465.76	2931.52
Leq23-01h at max. façade	2944.18	2983.55	0.19	0.11	-1466.09	2932.18
Estimated bedroom indoor LNight	2956.67	2996.03	0.17	0.11	-1472.33	2944.67
Leq05-06h at min. façade	3057.73	3097.09	0.10	0.07	-1522.86	3045.73
Leq24 at min. façade	3057.91	3097.27	0.10	0.07	-1522.95	3045.91
Leq06-07h at min. façade	3057.92	3097.28	0.10	0.07	-1522.96	3045.92
Lden at min. façade	3057.94	3097.30	0.10	0.07	-1522.97	3045.94
LDay at min. façade	3058.00	3097.36	0.10	0.07	-1523.00	3046.00
LNight at min. façade	3058.04	3097.40	0.10	0.07	-1523.02	3046.04
Leq19-23h at min. façade	3058.16	3097.53	0.10	0.07	-1523.08	3046.16
Leq01-05h at min. façade	3058.54	3097.90	0.10	0.07	-1523.27	3046.54
Leq23-01h at min. façade	3058.92	3098.29	0.10	0.07	-1523.46	3046.92

Railway noise:

Exposure indicator	AIC	BIC	M-Z R^2	NK R^2	Log-Likelihood	Deviance
Lden at max. façade	1756.57	1793.6	0.41	0.28	-872.28	1744.57
Leq24 at max. façade	1756.95	1793.99	0.41	0.28	-872.48	1744.95
Leq19-23h at max. façade	1757.44	1794.48	0.41	0.28	-872.72	1745.44
Leq23-01h at max. façade	1757.55	1794.58	0.41	0.28	-872.77	1745.55
Leq05-06h at max. façade	1757.72	1794.75	0.41	0.28	-872.86	1745.72
LNight at max. façade	1758.14	1795.18	0.41	0.28	-873.07	1746.14
LDay at max. façade	1758.42	1795.46	0.41	0.28	-873.21	1746.42
Leq06-07h at max. façade	1759.45	1796.49	0.41	0.28	-873.73	1747.45
Leq01-05h at max. façade	1781.93	1818.96	0.39	0.27	-884.96	1769.93
Leq05-06h at min. façade	1882.15	1919.18	0.30	0.22	-935.07	1870.15
Leq24 at min. façade	1882.65	1919.69	0.30	0.22	-935.33	1870.65
Lden at min. façade	1882.98	1920.02	0.30	0.22	-935.49	1870.98
Leq06-07h at min. façade	1883.00	1920.04	0.30	0.22	-935.50	1871.00
Leq23-01h at min. façade	1883.39	1920.43	0.30	0.22	-935.70	1871.39
Leq19-23h at min. façade	1883.62	1920.66	0.30	0.22	-935.81	1871.62
LDay at min. façade	1883.83	1920.87	0.30	0.21	-935.92	1871.83
LNight at min. façade	1884.84	1921.88	0.30	0.21	-936.42	1872.84
Leq01-05 at min. façade	1913.35	1950.38	0.29	0.20	-950.67	1901.35

Aircraft noise:

Exposure indicator	AIC	BIC	M-Z R^2	NK R^2	Log-Likelihood	Deviance
LNight	1744.41	1780.3	0.32	0.25	-866.21	1732.41
Lden	1792.03	1827.92	0.31	0.22	-890.02	1780.03
Leq01-05h	1792.79	1828.68	0.24	0.22	-890.39	1780.79
Leq19-23h	1816.10	1851.98	0.30	0.21	-902.05	1804.10
Leq23-01h	1819.11	1855.00	0.28	0.21	-903.56	1807.11
Leq06-07h	1824.50	1860.38	0.28	0.21	-906.25	1812.50
Leq24	1847.31	1883.19	0.27	0.19	-917.65	1835.31
LDay	1863.93	1899.81	0.26	0.18	-925.96	1851.93
Leq05-06h	1908.17	1944.05	0.19	0.16	-948.08	1896.17

B) Supplementary Figures

Figure S1

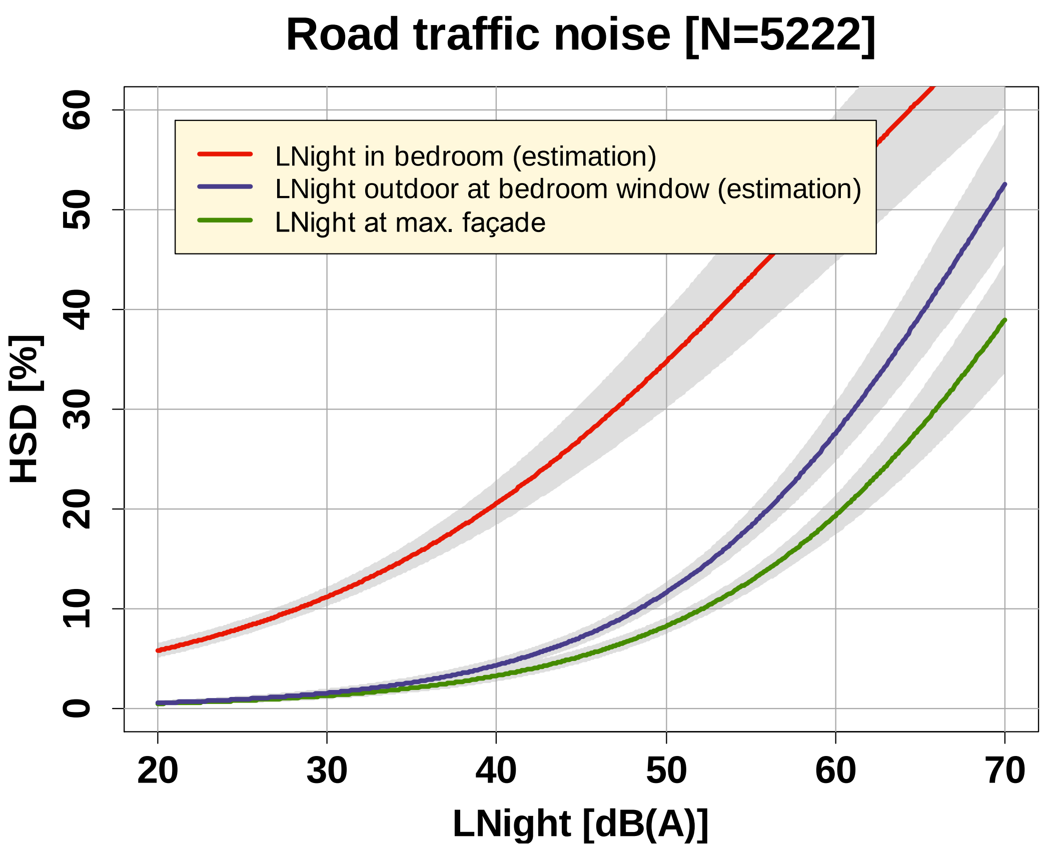

Figure S1: %HSD as function of road traffic noise (LNight) in the bedroom (red curve), outdoor at the bedroom window (blue curve), and at the maximum façade point (green curve). The curves are based on the adjusted model predictor set with covariates centered on the mean and include 95% confidence intervals as shaded areas.
